# Supplementary material for: Topology-transformable block copolymers based on a rotaxane structure: change in bulk properties with same composition
Source: Nat Commun. 2021 Oct 26;12:6175. doi: 10.1038/s41467-021-26249-0 (PMC8548399; doi:10.1038/s41467-021-26249-0)
Supplement: Supplementary file 1 — Supplementary Information [file 41467_2021_26249_MOESM1_ESM.pdf]

Supplementary Information

Topology-transformable block copolymers based on a rotaxane structure: change in bulk properties with same composition

Hiroki Sato,[1] Daisuke Aoki,[1] Hironori Marubayashi,[1] Satoshi Uchida,[1] Hiromitsu Sogawa,[1] Shuichi Nojima,[1] Xiaobin Liang,[1] Ken Nakajima,[1] Teruaki Hayakawa,[2] and Toshikazu Takata[1,3,4]

[1]Department of Chemical Science and Engineering, Tokyo Institute of Technology, Ookayama, Meguro, Tokyo 152-8552, Japan. [2]Department of Materials Science and Engineering, Tokyo Institute of Technology, Ookayama, Meguro, Tokyo 152-8552, Japan. [3]JST-CREST, Ookayama, Meguro, Tokyo 152-8552, Japan. [4]Graduate School of Advanced Science and Engineering, Hiroshima University, Kagamiyama, Higashi-Hiroshima, Hiroshima 739-8527, Japan

Table of Contents

Supplementary Methods--S2-S29

Materials and instruments --S2-S3

1) Topology transformable ABC star polymer ---S4-S18

1-1) Synthesis of ABC star polymer connected by rotaxane structure-----------------S4-S10

1-2) Topology transformation of ABC star polymer -------------------------------------S11-S18

2) Topology transformable A_2B_2 star polymer ---S19-S29

2-1) Synthesis of A_2B_2 star polymer connected by rotaxane structure -------------S19-S22

2-2)Topology transformation from A_2B_2-star to ABA-linear---------------------------S23-S27

Supplementary Discussion--S28-S29

Model polymer---S28-S29

Supplementary References--S29

Supplementary Methods

Materials

Dichloromethane was purchased from ASAHI GLASS CO., LTD., and distilled over CaH_2 under a nitrogen atmosphere after being washed with water. δ-Valerolactone [99%, Tokyo Chemical Industry Co., Ltd. (TCI)] was distilled over CaH_2 under reduced pressure. Styrene was used after passing through alumina column. Copper(I) bromide [>99%, Wako Pure Chemical Industries, Ltd. (Wako)] was used after washing with conc. hydrobromic acid. Diphenyl phosphate (99%, TCI), 3,5-dimethylphenyl isocyanate (98%, TCI), 2,2'-azodiisobutyronitrile (AIBN) (>98%, TCI), monohydroxy-terminated PDMS (M_w = 10000, Gelest, Inc.), *N*,*N*,*N*',*N*'',*N*''-pentamethyldiethylenetriamine (PMDETA) (99%, TCI), 4-hydroxy-3,5-dimethylbenzaldehyde (>98%, TCI), 1,6-dibromohexane (>97%, TCI), and acetic anhydride (98%, SIGMA-ALDRICH) were used as received. **Axle 1**[1], **wheel**[1], and **azide-terminated PDMS**[2] were synthesized according to the literatures. Other commercially available regents and solvents were used as received.

Instruments

^{1}H- (400 MHz) and ^{13}C (100 MHz) NMR spectra were recorded on a JEOL AL-400 spectrometer and ^{1}H- (300 and 500 MHz) and ^{13}C (125 MHz) NMR spectra were recorded on a Bruker spectrometer, with tetramethylsilane (TMS) as an internal standard. IR spectra were recorded on a JASCO FT/IR-230 spectrometer. FAB and ESI HR-MS spectra were obtained at the Center for Advanced Materials Analysis, Tokyo Institute of Technology on request. The size exclusion chromatography (GPC) was performed at 30 °C in $CHCl_3$ (0.85 mL / min) using a JASCO PU-2080 system equipped with a set of a Shodex K-804 and a Shodex K-805 columns. The number average molecular weight (M_n), weight average molecular weight (M_w), and polydispersity index (M_w/M_n) of the polymers were calculated on the basis of a polystyrene calibration. Preparative GPC was carried out using an HPLC LC-918 instrument by Japan Analytical Industry with a Megapak-Gel 201C. MALDI-TOF-MS was taken on a Shimadzu AXIMA-CFR mass spectrometer. The spectrometer was equipped with a nitrogen laser (λ = 337 nm) and with pulsed ion extraction. The operation was performed at an accelerating potential of 20 kV by a linear-positive ion mode. The sample polymer solution (1 mg / mL) was prepared in $CHCl_3$, and the matrix, dithranol, cationizing agent, and sodium trifluoroacetate, were dissolved in $CHCl_3$ or THF (10 and 1 mg / mL, respectively). The polymer solution and the matrix solution were mixed, and 1 µL portion of the mixed solution was deposited onto a sample target plate and allowed to dry in the air at room temperature. Mass values were calibrated by the two-point method with insulin β plus H^+ at

3497.96 and *R*-cyanohydroxycinnamic acid dimer plus H^+ at 379.35. Thermal analysis was carried out using a Shimadzu DSC-60 under a nitrogen gas atmosphere. Bruker NANOSTAR (λ = 0.1542 nm) with 2-dimensional detector, extended sample chamber, stage for grazing incidence SAXS (GISAXS) measurements, and lab-made heating system was used for A_2B_2 polymer system. Synchrotron small-angle X-ray scattering (SR-SAXS) and wide-angle X-ray diffraction (SR-WAXD) simultaneous measurements were performed at the beam lines BL-6A and 10C (λ = 0.1500 and 0.1488 nm, respectively) of the Photon Factory in KEK (Tsukuba, Japan). Two-dimensional SAXS patterns were detected using PILATUS3 1M and 2M (DECTRIS Ltd.) at BL-6A and 10C, respectively. Two-dimensional WAXD patterns were detected using PILATUS 100K and 200K (DECTRIS Ltd.) at BL-6A and 10C, respectively. Samples were kept at constant temperatures (25, 70, and 120 °C) during the measurements. A series of X-ray structure analyses were performed using handmade software.[3,4] The specimen was ultramicrotomed to a thickness of 70–90 nm using a LEICA EM FC7 at −160 °C. Hitachi SU9000 operating at 30 kV was used in the bright-field STEM (BF-STEM) mode to observe the phase-separated structure in ultramicrotomed films (unstained). The energy-dispersive X-ray spectroscopy (EDS) map in the same viewing field as BF-STEM was obtained by an AMETEK EDS analyzer incorporated in SU9000. TEM images of ultramicrotomed films (stained with RuO_4) were observed by a JEOL JEM-1010BS at 100 kV. The viscoelastic properties were obtained by dynamic mechanical analysis (Rheometers, AR 2000ex, TA Instruments, USA).

1) Topology transformable ABC star polymer

1-1) Synthesis of ABC star polymer connected by rotaxane structure

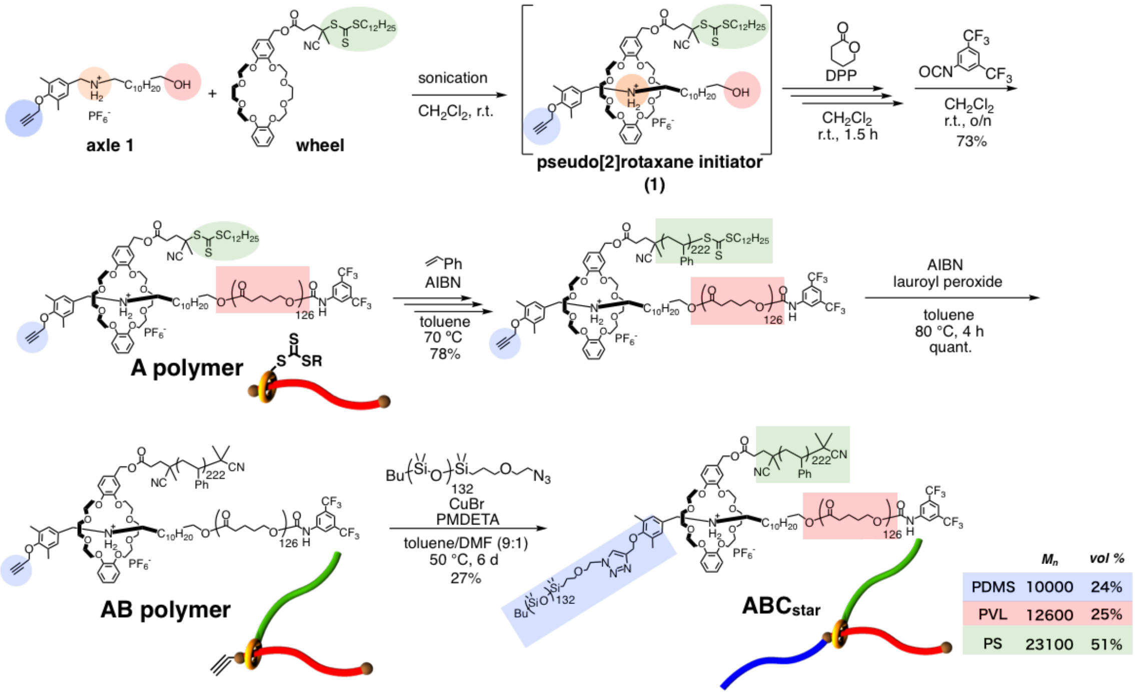

<u>Synthesis of **A polymer**</u>

A mixture of the axle component **axle 1** (29 mg, 0.055 mmol) and a wheel component **Wheel** (56 mg, 0.062 mmol) in dry CH_2Cl_2 (1.4 mL) was sonicated at ambient temperature until the solution got transparent to afford a solution of the pseudo[2]rotaxane initiator (**1**). Diphenyl phosphate (DPP) (14 mg, 0.055 mmol) and δ-VL (0.5 mL, 5.5 mmol) were then added to the mixture under an argon atmosphere. After 1.5 h, 3,5-bis(trifluoromethyl)phenyl isocyanate (0.086 mL, 0.5 mmol) was added to the resulting mixture and stirred overnight. The mixture was poured into a mixed solvent (ethanol/hexane = 1/9 (v/v)) to obtain a crude macromolecular [2]rotaxane. Purification by preparative GPC yielded **A polymer** (PVL_{126}, 579 mg, 73%) as a yellow solid.

Polymerization degree of the PVL was calculated by ^1H NMR using the integrals of signal e (5.03 ppm) and A (2.42-2.26 ppm).

^{1}H-NMR (300 MHz, $CDCl_3$): 7.99 (2H, s), 7.72 (1H, s), 7.53 (1H, s), 7.23–6.80 (9H, m, aromatics), 5.03 (2H, s), 4.47 (2H, m), 4.42 (2H, d, 2.4 Hzj), 4.26–4.00 (Polymer+α), 3.85 (8H, m), 3.70–3.46 (8H, m), 3.32 (2H, t, 7.8 Hz), 3.13 (2H, brs), 2.67 (2H, t, 7.8 Hz), 2.53 (1H, t, 2.4 Hz), 2.42–2.26 (Polymer), 2.13 (6H, s), 1.88 (3H, s), 1.78–1.56 (Polymer), 1.35–1.00 (alkyl), 0.88 (3H, t, 7.2 Hz).

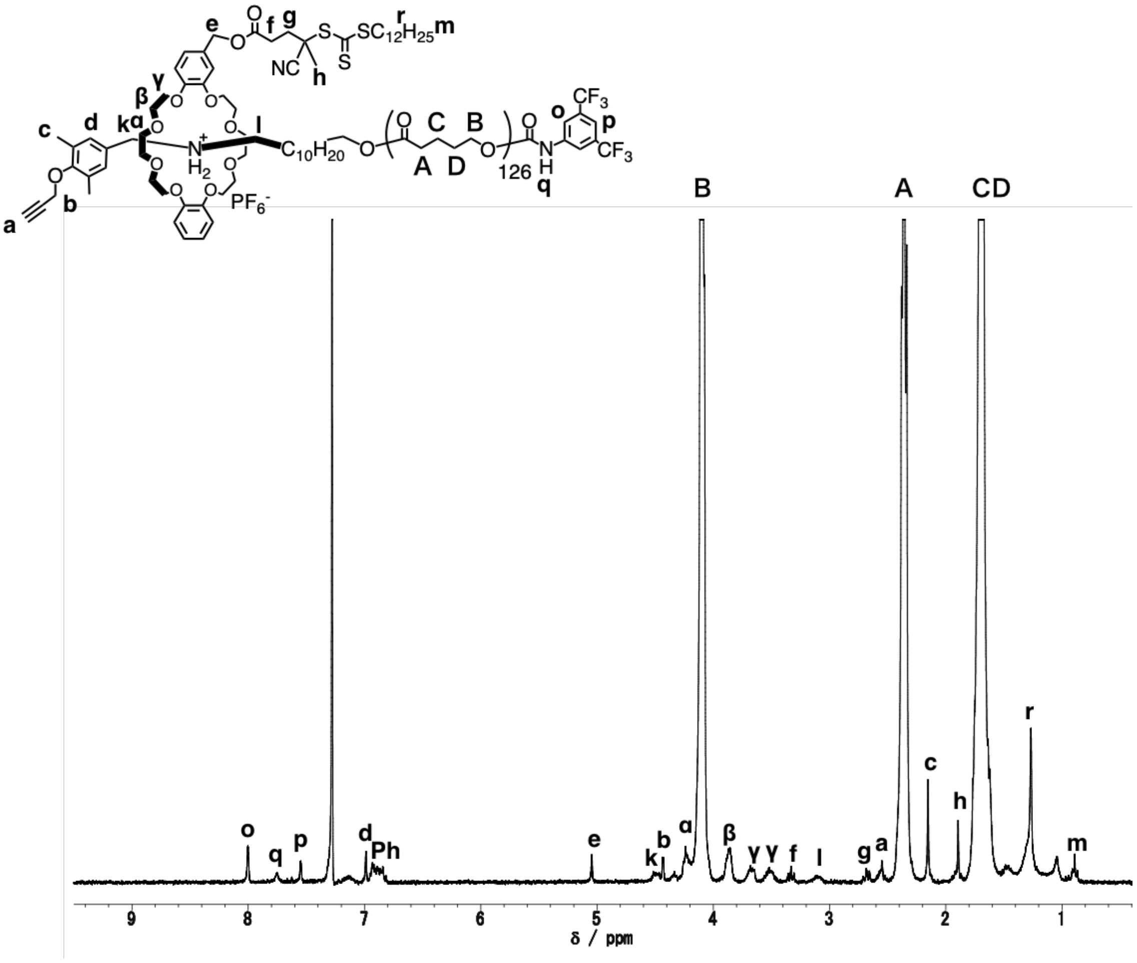

Supplementary Figure 1-1. ^{1}H NMR spectrum of the obtained **A polymer** (500 MHz, $CDCl_3$, 298 K).

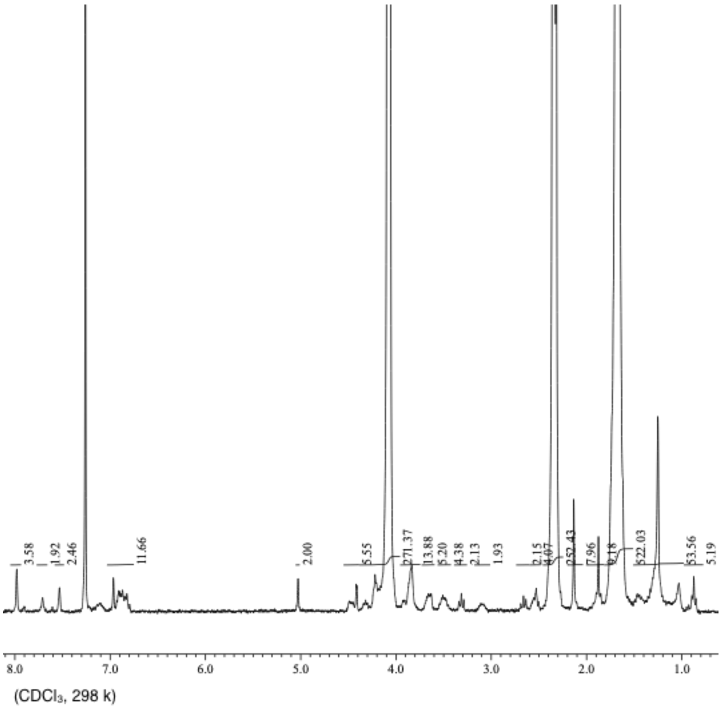

Supplementary Figure 1-2. ^{1}H NMR spectrum with integration values of the obtained **A polymer** (500 MHz, $CDCl_3$, 298 K).

<u>Synthesis of **AB polymer**</u>

To a solution of **A polymer** (290 mg, 0.020 mmol) in styrene (0.92 mL, 8 mmol) was added 0.20 mL of a solution of AIBN in toluene (8.2 mg in 5 mL, 2 µmol) and degassed three times by freeze-pump-thaw cycles. Then the system was purged with argon, closed and stirred at 70 °C. After 20 h, the polymerization was quenched via rapid cooling in liquid nitrogen and exposure to air. The polymer was isolated by reprecipitation into methanol to afford the crude product as a yellow solid. Finally, reprecipitation into diethyl ether yielded **AB polymer** with RAFT agent (PVL_{126}-*rot*-PS_{222}), 581 mg, 78% as a yellow solid.

Polymerization degrees of the **AB polymer** were calculated by ^{1}H NMR using the integrals of aromatic signal (7.10-6.29 ppm) and signal of PVL (2.38-2.30 ppm).

^{1}H-NMR (300 MHz, $CDCl_3$): 7.98 (2H, s), 7.67 (1H, brs), 7.54 (1H, s), 7.10–6.29 (PS aromatics), 4.95 (2H, br), 4.39 (2H, br), 4.25–4.02 (PVL —$COCH_2CH_2CH_2CH_2O$—), 3.84 (8H, br), 3.70–3.46 (8H, m), 3.24 (2H, br), 3.11 (2H, br, $ArCH_2NH_2^+CH_2$—), 2.50 (1H, brs), 2.38–2.30 (PVL —$COCH_2CH_2CH_2CH_2O$—), 2.12 (6H, brs), 2.0–1.2 (PVL+PS), 0.88 (3H, br).

Removal of RAFT moiety via radical coupling[5]

A solution of **AB polymer** with RAFT agent (581 mg, 0.016 mmol), AIBN (62 mg, 0.38 mmol) and lauroyl peroxide (15 mg, 0.038 mmol) in toluene (3 mL) was degassed three times by freeze-pump-thaw cycles. Then the system was closed and heated at 80 °C. After four hours, the reaction was quenched via rapid cooling with liquid nitrogen and exposure to air. The polymer was isolated by reprecipitation into ether/toluene (9/1) to afford **AB polymer** (581 mg, quant.) as a white solid.

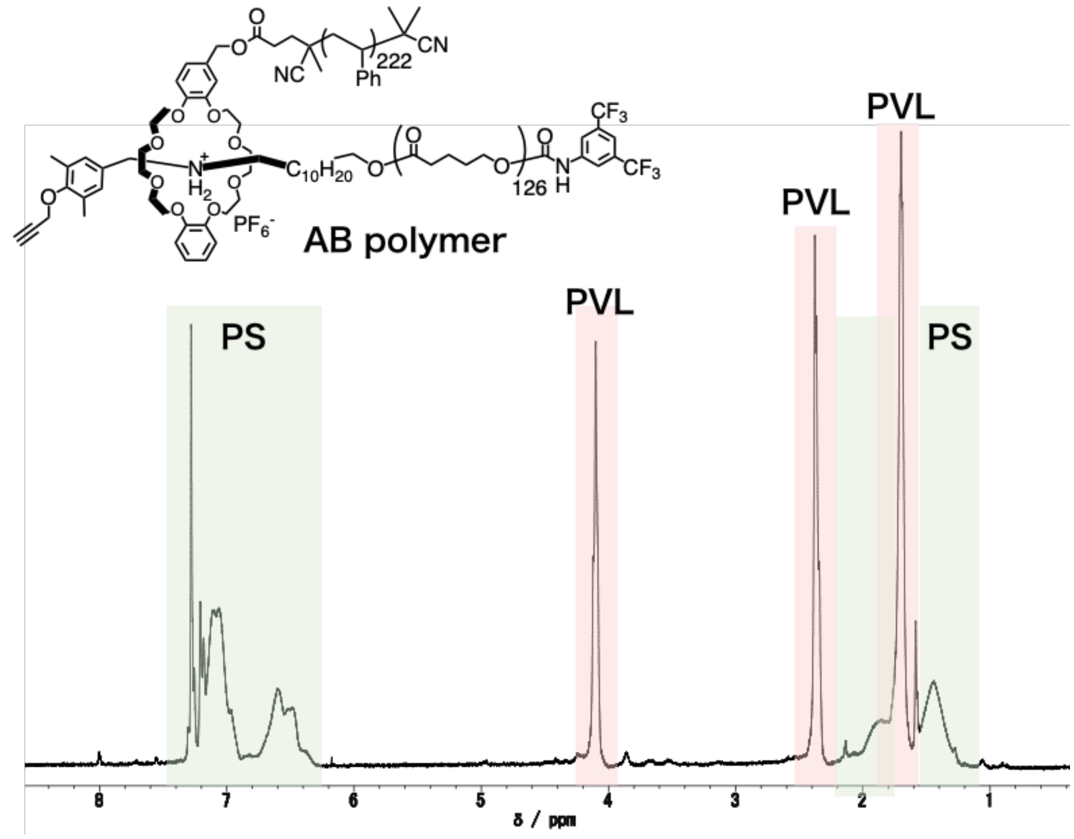

Supplementary Figure 2-1. ^{1}H NMR spectrum of the obtained **AB polymer**

(500 MHz, $CDCl_3$, 298 K).

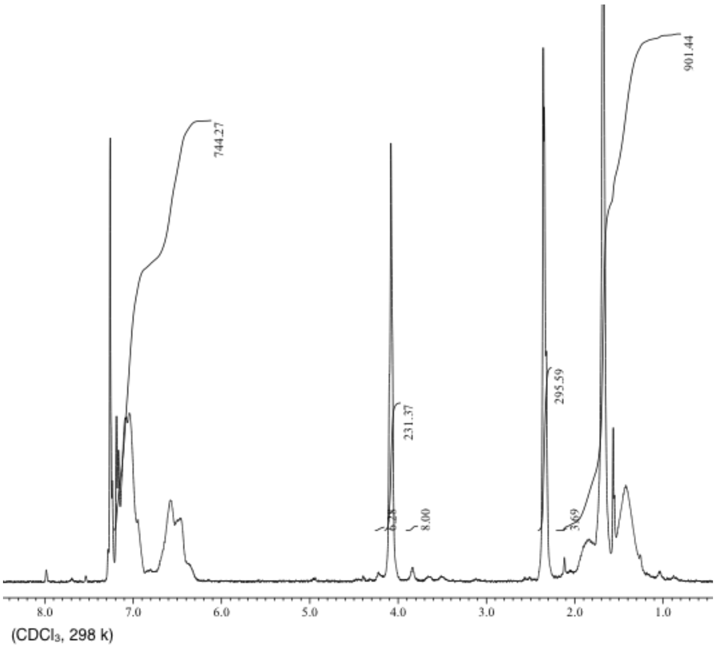

Supplementary Figure 2-2. ^{1}H NMR spectrum with integration values of the obtained **AB polymer** (500 MHz, $CDCl_3$, 298 K). The polymer composition (ratio of A and B) is not the same as ABC star polymers, i.e., 12600(PVL):23100(PS):10000(PDMS), because synthesis of ABC star polymers went through purification process which changed polymer composition. To remove an excess amount of PDMS (C) after synthesis of ABC polymer, preparative GPC was applied several times. This purification process also removed the ABC polymers with relatively low molecular weight, especially whose PS (B polymer) length is short, resulting in the increase of PS fraction in ABC polymers.

Synthesis of PDMS-N_3[2]

Synthesized according to the literature with a monohydroxy-terminated PDMS (M_w = 10000) and purified by reprecipitation into methanol.

Synthesis of **ABC$_{star}$**[1]

In a dried 25 mL Schlenk tube, **AB polymer** (553 mg, 0.015 mmol), PDMS-N_3 (380 mg, 0.038 mmol), and toluene (10 mL) were placed and degassed twice by freeze-pump-thaw cycles. In a vial, CuBr (5.5 mg, 0.019 mmol), PMDETA (7.9 µL, 0.019 mmol), and DMF (50 µL) were placed and degassed by Ar bubbling, to make a catalyst solution. The catalyst solution was added to the Schlenk tube under Ar atmosphere. Then the tube was sealed and heated at 50 °C. After 1 day and 3 days, the catalyst solution was added again. After 5 days heating, the reaction was

quenched by adding NH_4PF_6 aq. (50 mg / 10 mL) and extracted with chloroform three times. The crude polymer was exposed to the click reaction conditions again. Finally, purification with preparative GPC (column: YMC-GPC T-60000) yielded **ABC$_{star}$** (189 mg, 27%) as a colorless solid.

^{1}H-NMR (500 MHz, $CDCl_3$): 7.98 (2H, s), 7.53 (1H, s), 7.10–6.29 (PS aromatics), 4.25–4.02 (PVL —$COCH_2CH_2CH_2C$*H_2*O—), 2.38–2.30 (PVL —COC*H_2*$CH_2CH_2CH_2O$—), 2.13 (6H, brs), 2.0–1.2 (PVL+PS), 0.07 (PDMS).

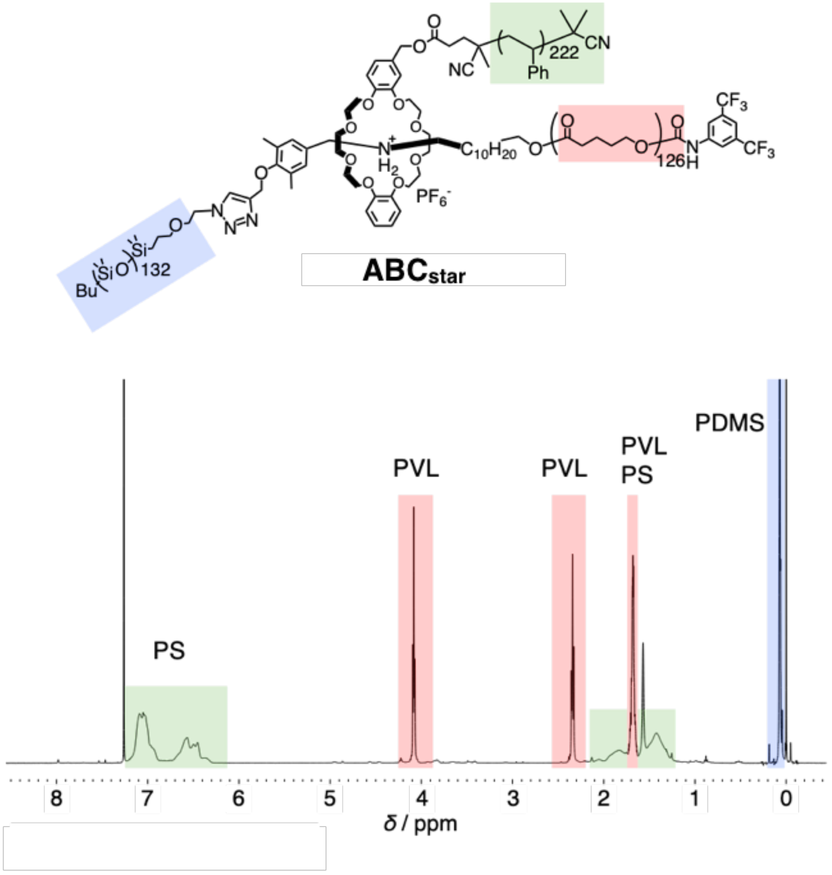

Supplementary Figure 3-1. ^{1}H NMR spectrum of the obtained **ABC$_{star}$** (500 MHz, $CDCl_3$, 298 K).

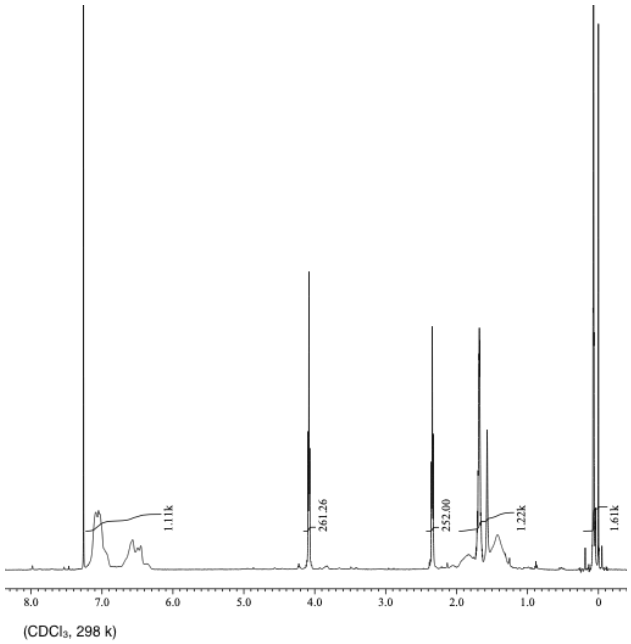

Supplementary Figure 3-2. ^{1}H NMR spectrum with integration values of the obtained **ABC$_{star}$** (500 MHz, $CDCl_3$, 298 K).

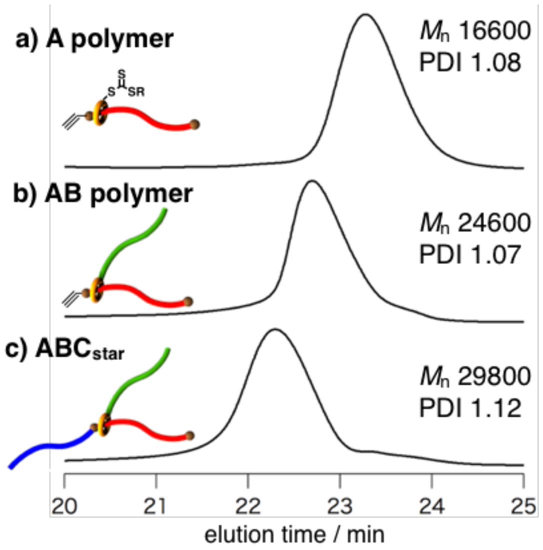

Supplementary Fig. 4. GPC traces of the obtained polymers; a) **A polymer**, b) **AB polymer**, and c) **ABC$_{star}$** ($CHCl_3$, RI, PS standards).

1-2) <u>Topology transformation of ABC star polymer</u>

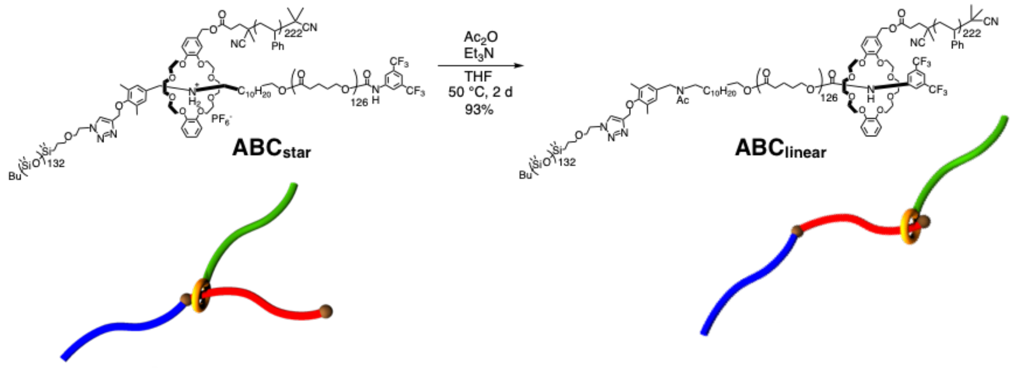

<u>Topology transformation of rotaxane-linked ABC terpolymer from star to linear</u>

A solution of **ABC$_{star}$** (144 mg, 3.7 µmol), acetic anhydride (19 µL, 0.19 mmol) and triethylamine (50 µL, 0.37 mmol) in THF (2 mL) was stirred at 50 °C under argon atmosphere. After two days, the reaction was quenched by addition of water. Then, the mixture was extracted with chloroform four times, followed by passing through $MgSO_4$ column and evaporated to afford a crude polymer as a reddish solid. Purification by preparative GPC followed by reprecipitation into cold hexane yielded **ABC$_{linear}$** (136 mg, 93%) as a colorless solid.

^{1}H-NMR (500 MHz, 298 K, $CDCl_3$): 8.76 (H, br, —N*H*COAr), 7.90 (3H, br), 7.3–6.3 (PS aromatics), 4.3–4.0 (PVL —$COCH_2CH_2CH_2CH_2O$—), 2.4–2.3 (PVL —$COCH_2CH_2CH_2CH_2O$—), 2.0–1.2 (PVL+PS), 0.3–0.0 (PDMS).

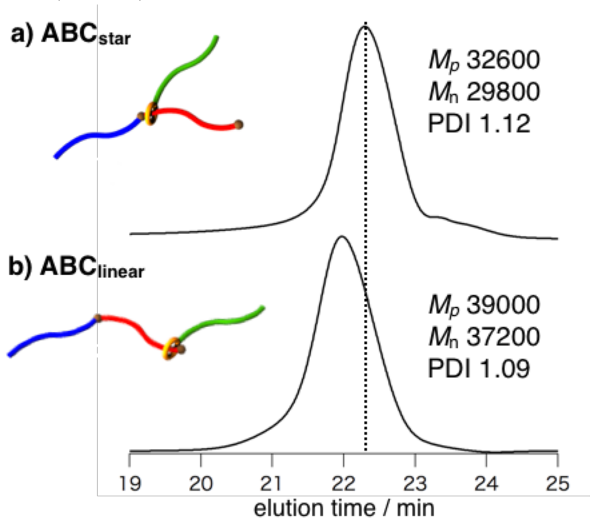

Supplementary Figure 5. GPC traces through the star/linear topology transformation; a) **ABC$_{star}$** and b) **ABC$_{linear}$** ($CHCl_3$, RI, PS standards).

^{1}H-NMR

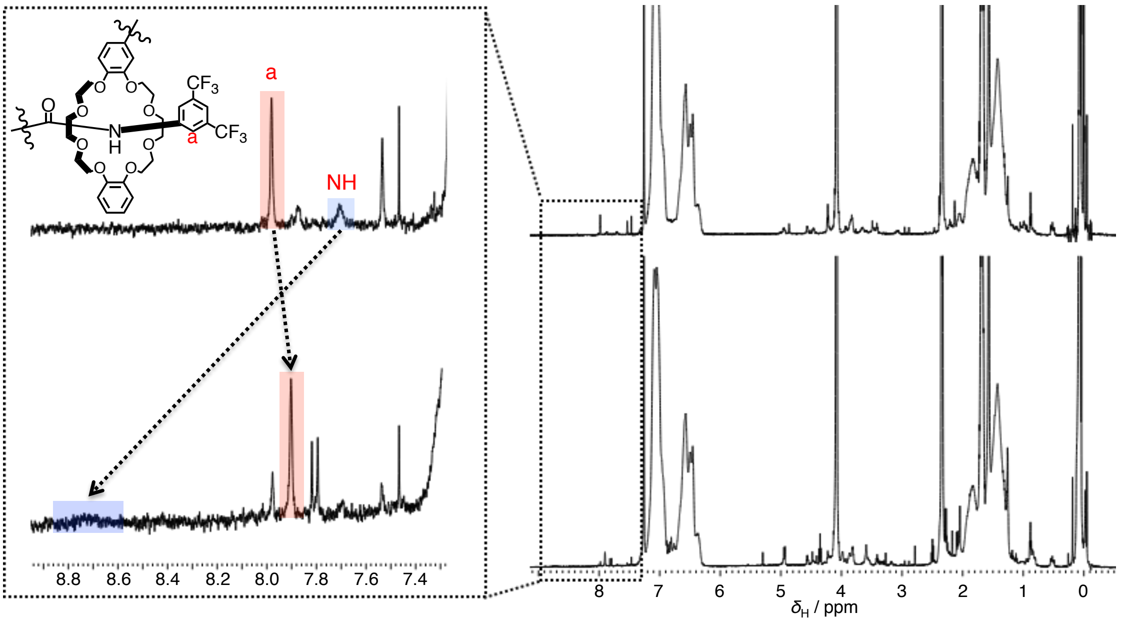

Supplementary Figure 6-1. ^{1}H-NMR spectra of **ABC$_{star}$** (upper) and **ABC$_{linear}$** (lower) (500 MHz, $CDCl_3$, 298 K).

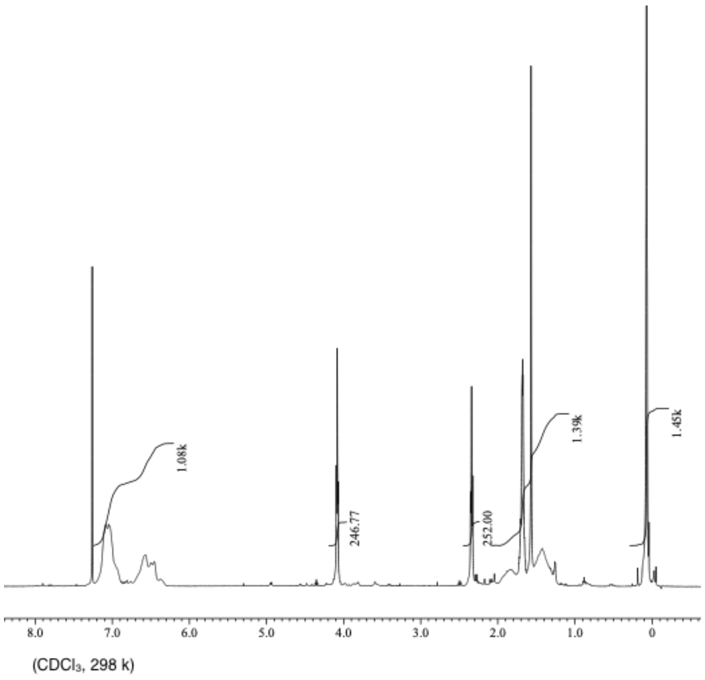

Supplementary Figure 6-2. ^{1}H NMR spectrum with integration values of the obtained **ABC$_{linear}$** (500 MHz, $CDCl_3$, 298 K).

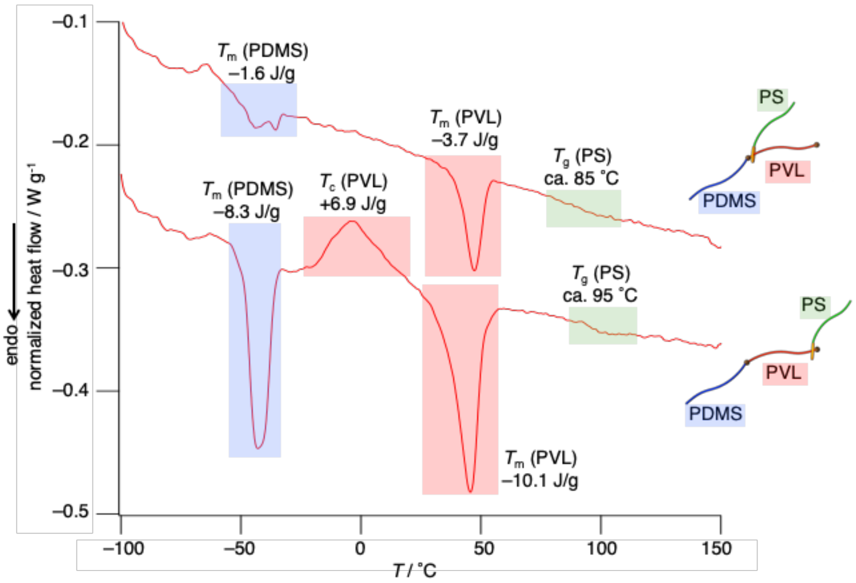

Supplementary Figure 7. DSC curves of **ABC$_{star}$** and **ABC$_{linear}$** (2nd heating, 10 °C/min).

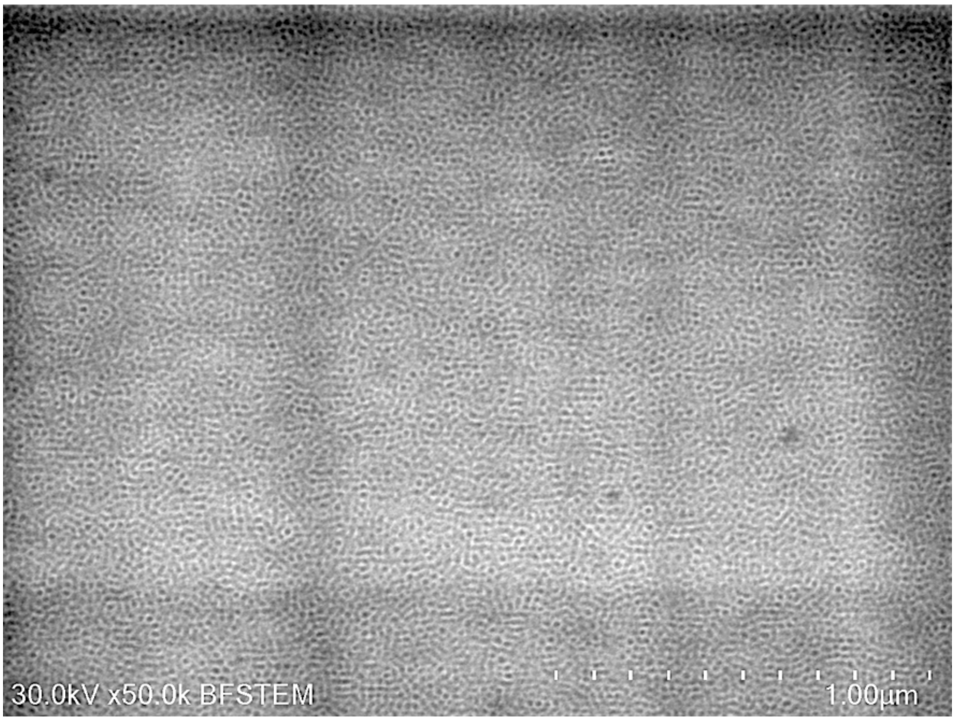

Supplementary Figure 8. BF-STEM image of **ABC$_{star}$** (unstained, 30 kV). Dark regions contain PDMS blocks.

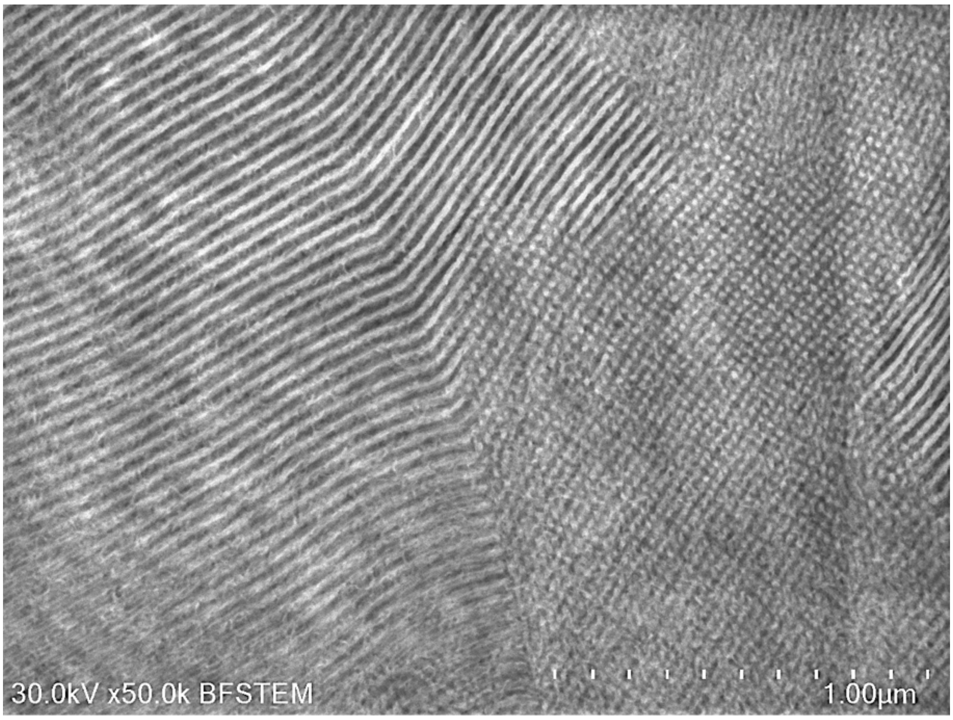

Supplementary Figure 9. BF-STEM image of **ABC$_{linear}$** (unstained, 30 kV). Dark regions contain PDMS blocks.

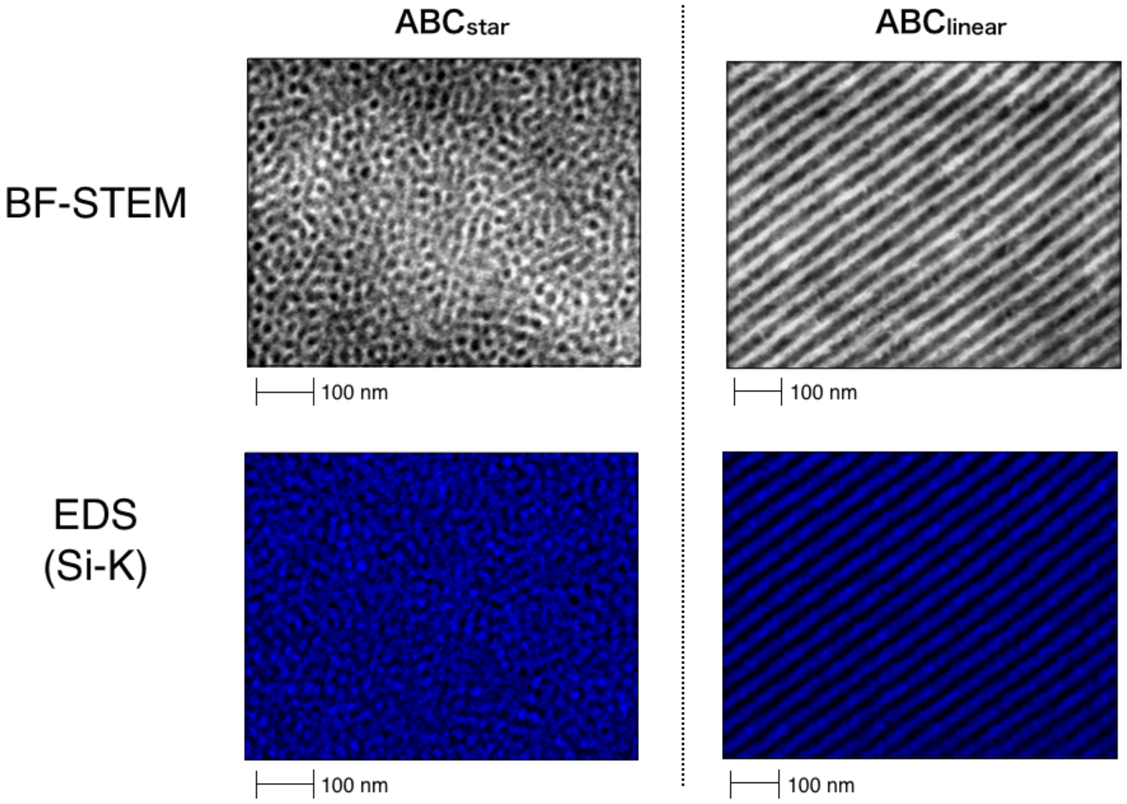

Supplementary Figure 10. STEM and EDS images of **ABC$_{star}$** and **ABC$_{linear}$**. EDS map of each polymer was obtained in the same viewing field as STEM.

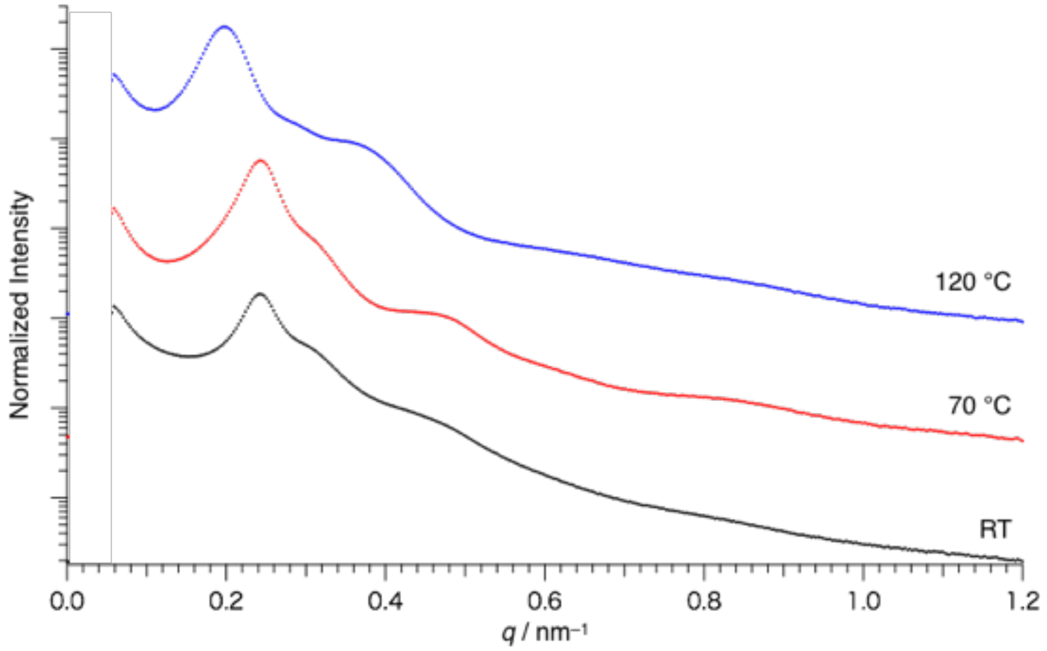

Supplementary Figure 11. SR-SAXS profiles of **ABC**$_{\mathbf{star}}$ at various temperatures.

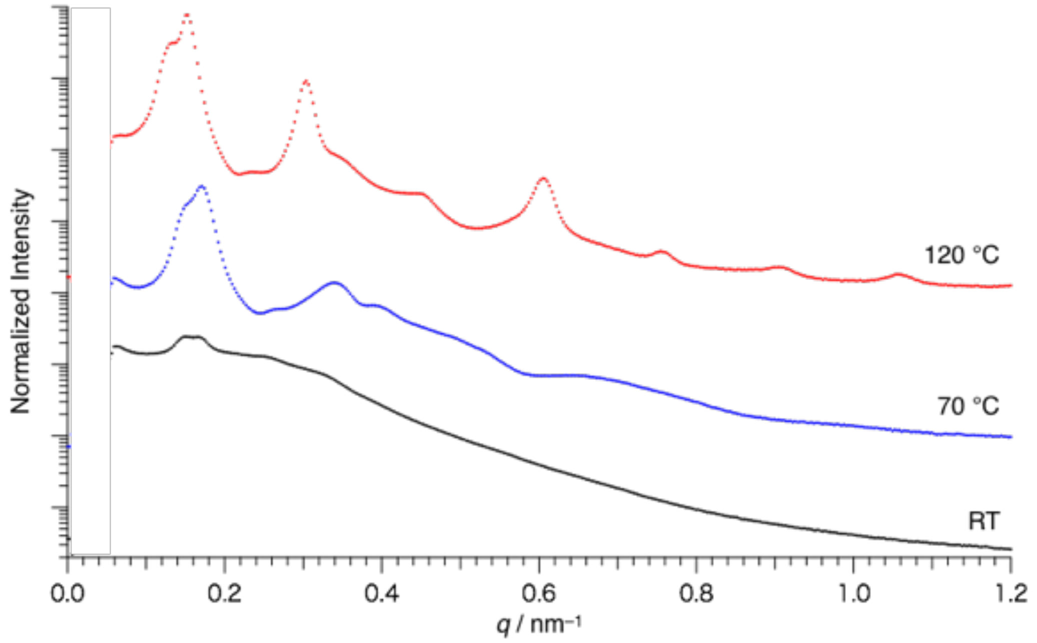

Supplementary Figure 12. SR-SAXS profiles of **ABC**$_{\mathbf{linear}}$ at various temperatures.

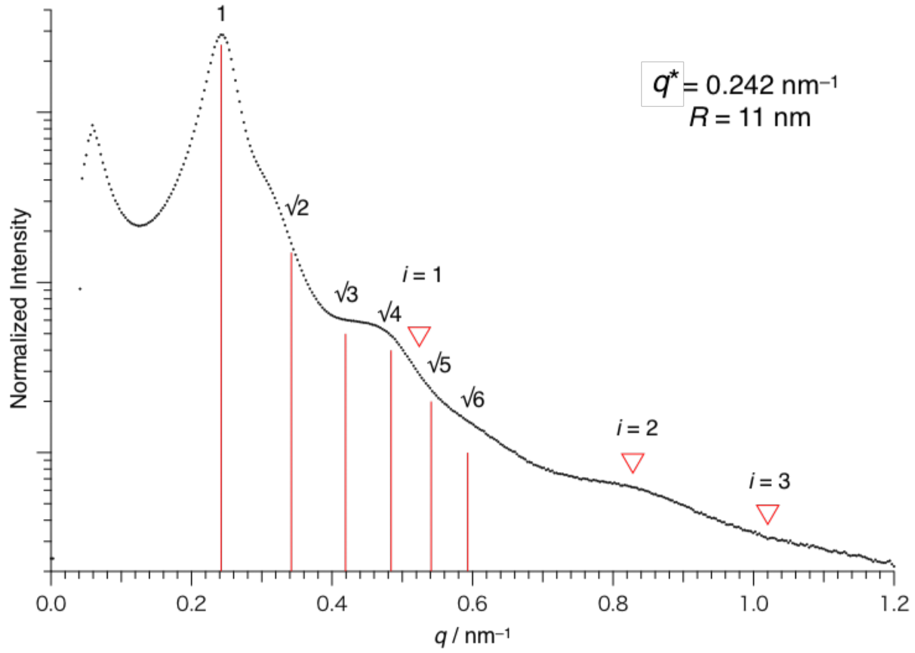

Supplementary Figure 13. Assigned SR-SAXS profile of **ABC$_{\mathbf{star}}$** (70 °C). The positions of scattering peaks derived from sphere structure (structure factor; # and red lines) and isolated sphere (form factor; *i* = # and red triangles) are indicated.

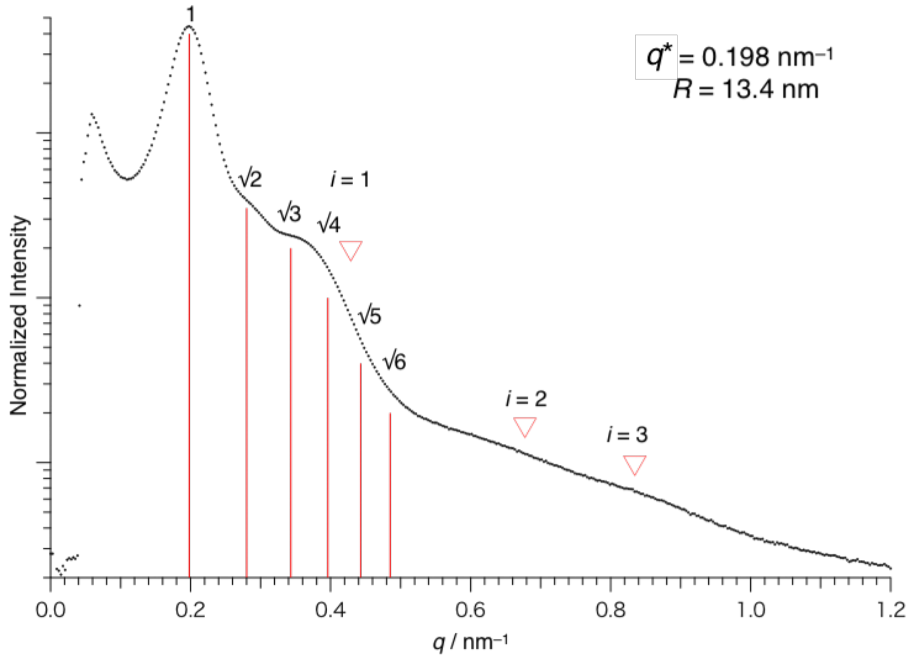

Supplementary Figure 14. Assigned SR-SAXS profile of **ABC$_{\mathbf{star}}$** (120 °C). The positions of scattering peaks derived from sphere structure (structure factor; # and red lines) and isolated sphere (form factor; *i* = # and red triangles) are indicated.

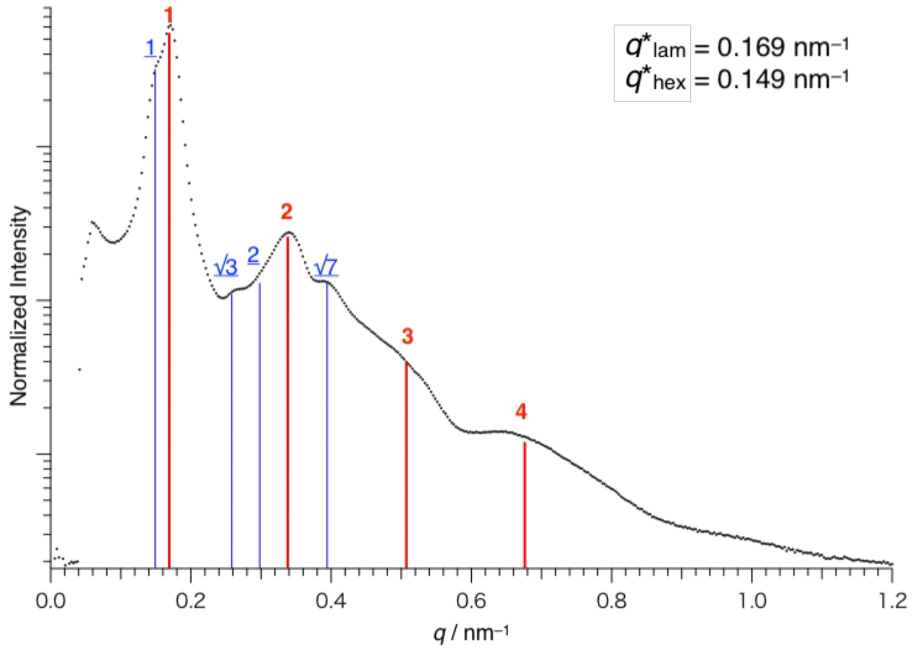

Supplementary Figure 15. Assigned SR-SAXS profile of **ABC**$_{\mathbf{linear}}$ (70 °C).

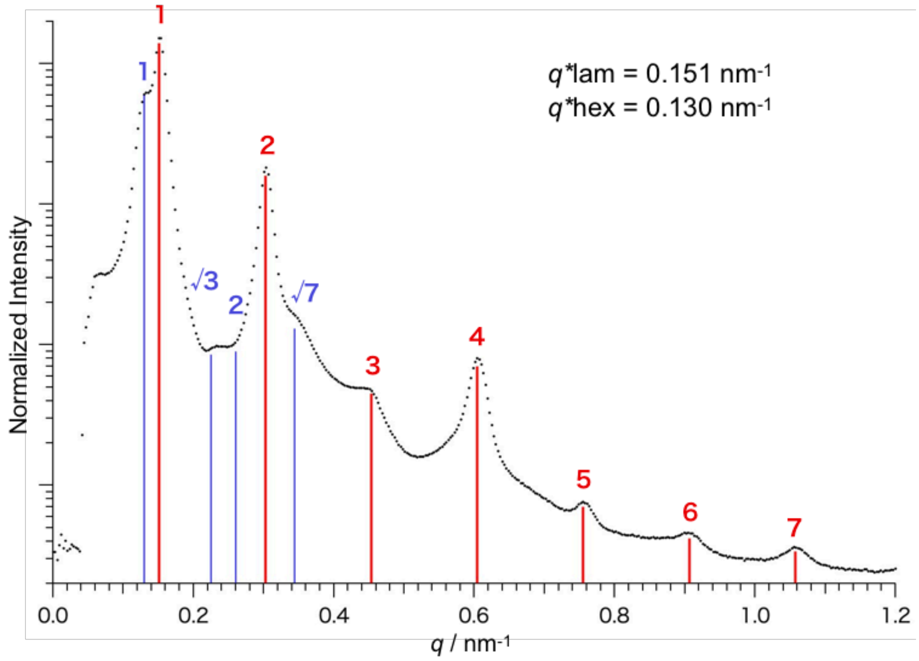

Supplementary Figure 16-1. Assigned SR-SAXS profile of **ABC**$_{\mathbf{linear}}$ (120 °C).

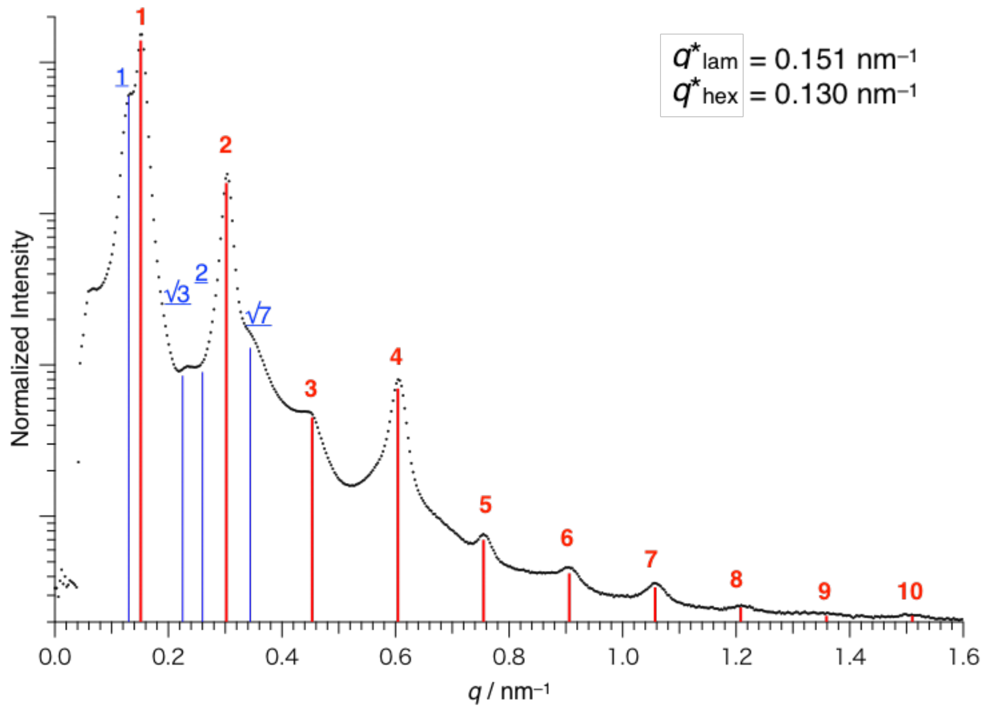

Supplementary Figure 16-2. Assigned SR-SAXS profile of **ABC$_{linear}$** in a wide q range (120 °C).

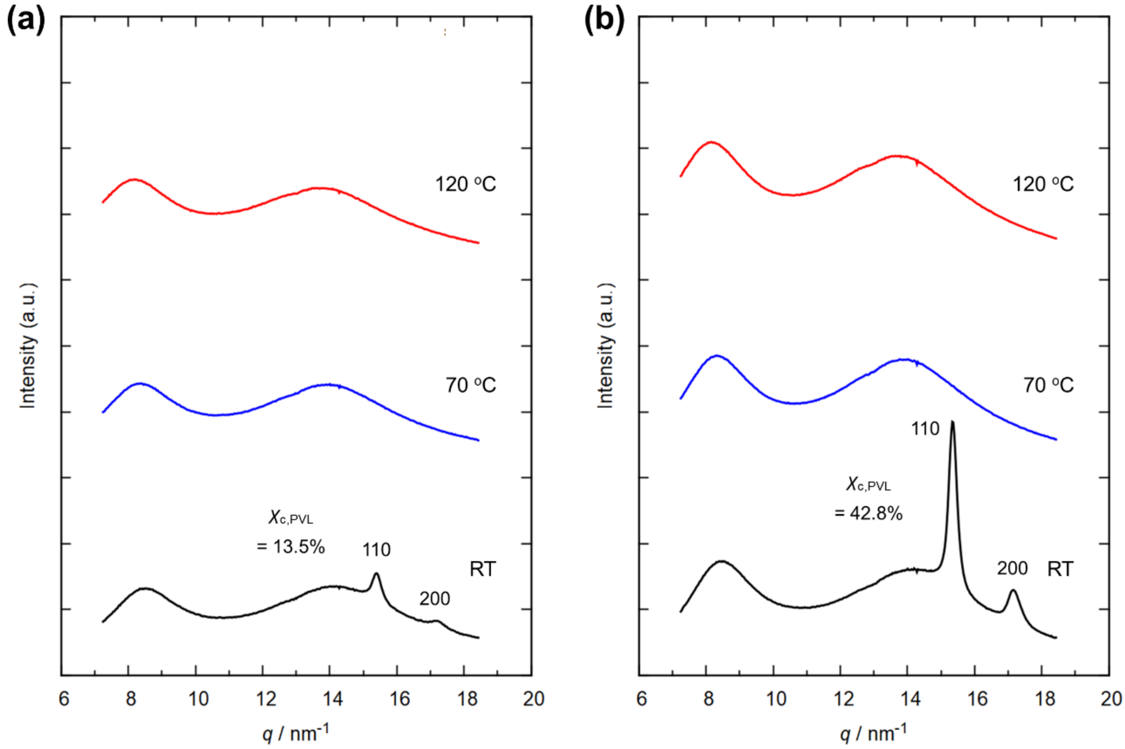

Supplementary Figure 17. SR-WAXD profiles at various temperatures of (a) **ABC$_{star}$** and (b) **ABC$_{linear}$**.

2) Topology transformable A_2B_2 star polymer

2-1) Synthesis of A_2B_2 star polymer connected by rotaxane structure

Synthesis of monomer (MeVL)[6]

In a 2 L 3-neck flask equipped with a mechanical stirrer, a dropping funnel, a thermometer, 3-methyl-1,5-pentanediol (16.5 g, 140 mmol), TEMPO (451 mg, 2.9 mmol), KBr (2.1 g, 18 mmol), tetrabutylammonium bromide (2.5 g, 7.8 mmol), H_2O (20 mL) and dichloromethane (500 mL) were placed and cooled to 0 °C. Then a mixture of ca. 10% NaOCl aq. (500 mL) and $NaHCO_3$ (17 g) was added dropwise over 1 h, followed by addition of TEMPO (250 mg), and the mixture was stirred vigorously at 0 °C. After 17 h, the organic phase was separated and the aqueous phase was extracted with dichloromethane twice. The combined organic phase was washed with sat. $Na_2S_2O_3$ aq. and brine, dried over $MgSO_4$, and evaporated, to yield a crude product (24.5 g) as a red liquid. Vacuum distillation over CaH_2 with a Vigreux column to yield the titled compound (12.7 g, 80%) as a pale yellow liquid. Colorless liquid was obtained by repeating distillation.
b.p. 67.8-69.1 °C (1 mmHg). ^{1}H-NMR (500 MHz, $CDCl_3$): 4.45–4.39 (H, m), 4.30–4.23 (H, m), 2.72–2.64 (H, m), 2.16–2.05 (2H, m), 1.95–1.89 (H, m), 1.57–1.48 (H, m), 1.07 (3H, d, 6.4 Hz). ^{13}C-NMR (125 MHz, $CDCl_3$): 171.2, 68.6, 38.3, 30.7, 26.6, 21.5.

Synthesis of **axle 2**

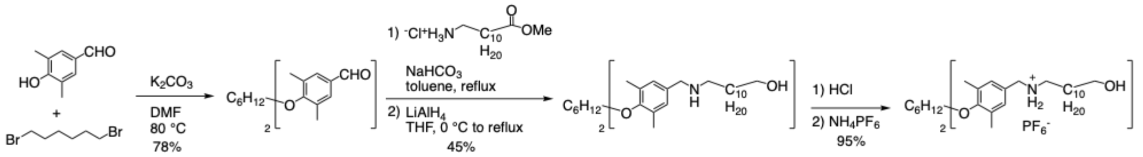

2 3 **axle 2**

Synthesis of **2**

To a mixture of dried K_2CO_3 (1.66 g, 12 mmol), 4-hydroxy-3,5-dimethylbenzaldehyde (1.08 g, 7.2 mmol) and DMF (16 mL) in a dried 50 mL round-bottom flask was added 1,6-dibromohexane (616 µL, 4.0 mmol), and the mixture was subsequently degassed by under light vacuum and refilling of the atmosphere by Ar (at least three cycles). Then the mixture was heated at 80 °C for 7 days. The reaction was quenched by adding water and the product was extracted with $CHCl_3$ (three times). The combined organic phase was washed with dilute HCl aq., sat. $NaHCO_3$ aq., water, brine, and dried over $MgSO_4$. Evaporation yielded 1.25 g of the crude product as a white solid. Recrystallization (EtOH) yielded the titled compound **2** (1.07 g, 78%).

^{1}H-NMR (500 MHz, $CDCl_3$): 9.87(2H, s), 7.55 (4H, s), 3.85 (4H, t, 6.6 Hz), 2.34 (12H, s), 1.92–1.87 (4H, m), 1.66–1.60 (4H, m). ^{13}C-NMR (125 MHz, $CDCl_3$): 191.60, 161.52 132.04, 131.88, 130.65, 72.26, 30.29, 25.96, 16.36.

Synthesis of **3**

In a 50 mL round-bottom flask equipped with Dean-Stark trap was placed **2** (1.07 g, 2.80 mmol), 12-methoxycarbonyldodecylammonium chloride (1.61 g, 6.07 mmol), $NaHCO_3$ (761 mg, 9.06 mmol), and toluene (ca. 20 mL). The mixture was refluxed for 7 h. Filtration followed by

evaporation yielded an imine compound (The consumption of the aldehyde was checked by ^{1}H-NMR). To a mixture of the imine and THF (15 mL) in a 50 mL two-neck flask was added $LiAlH_4$ (758 mg, 18.1 mmol) at 0 °C. Soon after the addition, the mixture was gelated. The mixture was stirred at 0 °C for 30 min and then refluxed for 2 h. The reaction was quenched by adding Rochelle salt soln. at 0 °C, then the product was extracted with $CHCl_3$ (4 times), followed by washing with Rochelle salt soln., brine, dried over $MgSO_4$, and evaporated, to yield 1.80 g of the crude product as a white solid. Purification by Si-column chromatography ($CHCl_3$/MeOH/Et_3N = 100/5/1) yielded 1.52 g of the product. The product was further purified by recrystallization (toluene/EtOH) to yield 953 mg (45%) of the titled compound **3**.

^{1}H-NMR (300 MHz, $CDCl_3$): 6.94 (4H, s), 3.76 (4H, t, 6.4 Hz), 3.65 (4H, s), 3.63 (4H, 6.6 Hz), 2.62 (4H, t, 7.3 Hz), 2.26 (12H, s), 1.84 (4H, t, 6.4 Hz), 1.9–1.2 (alkyl chain).
^{13}C-NMR (125 MHz, $CDCl_3$): 154.92, 135.42, 130.71, 128.50, 72.16, 62.93, 53.66, 49.63, 32.79, 30.36, 29.99, 29.55, 29.53, 29.52, 29.38, 27.33, 26.09, 25.72, 16.27.

Synthesis of **axle 2**

To a mixture of **3** (530 mg, 0.71 mmol), MeOH (3 mL), and THF (3 mL) was added 0.24 mL of 12 M HCl aq. The mixture was stirred at RT for 30 min. Then the solution was poured into excess amt. of ether, and the forming precipitate was collected. The precipitate was dissolved in MeOH (8 mL) and then NH_4PF_6/H_2O (985 mg / 4 mL, 6.0 mmol) was added slowly with stirring at RT. After 1 h, H_2O was added to form the precipitate. The titled compound was collected by filtration followed by washing with water and drying in vacuo (698 mg, 95%).

^{1}H-NMR (300 MHz, CD_3CN): 7.09 (4H, s), 6.62 (4H, brs), 4.02 (4H, brs), 3.80 (4H, t, 6.3 Hz), 3.47 (4H, dd, 11.5, 6.3 Hz), 2.45 (2H, t, 5.6 Hz), 2.27 (12H, s), 1.9–1.2 (alkyl chain).

^{13}C-NMR (100 MHz, DMSO-d_6): 156.17, 130.77, 130.37, 126.98, 71.75, 60.69, 49.70, 46.57, 32.53, 29.88, 29.11, 29.00, 28.97, 28.92, 28.78, 28.46, 25.88, 25.56, 25.52, 25.33, 15.97.

HR-MS(ESI$^+$, MeOH): $[M-2PF_6]^{2+}$ calcd. for $C_{48}H_{86}N_2O_4$, 377.3288; found, 377.3286.

Synthesis of **Linear polymer**

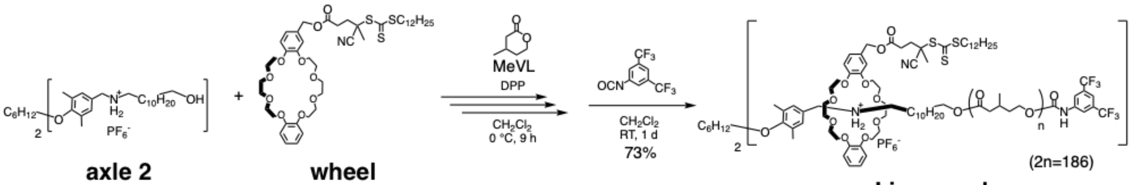

axle 2 **wheel** **Linear polymer**

A mixture of **axle 2** (16 mg, 0.016 mmol) and **wheel** (55 mg, 0.06 mmol) was dissolved in CH_2Cl_2 (0.9 mL) by sonication in a dried Schlenk tube. Then, **MeVL** (0.49 mL, 4.5 mmol) and DPP (5.9 mg, 0.024 mmol) were added at 0 °C and stirred for 9 h under Ar atmosphere. The conversion was monitored by ^{1}H-NMR. After polymerization, 3,5-bis(trifluoromethyl)phenyl isocyanate (0.05 mL, 0.3 mmol) was added and stirred at ambient temperature for 1 day. The crude polymer (ca. 340 mg, containing remained solvent) was obtained by pouring into iced hexane/EtOH (95/5) followed by centrifugation. Purification by preparative GPC yielded Linear polymer (279 mg, 73%) as yellow viscous oil.

^{1}H-NMR (500 MHz, $CDCl_3$): 8.02, 7.98 (s, 4H), 7.97, 7.88 (s, 2H), 7.53 (s, 2H), 7.09 (brs, 4H), 6.98 (s, 4H), 6.96–6.80 (m, 16H), 5.03 (s, 4H), 4.49–4.41 (m, 4H), 4.34–4.01 (m, 2nH, PmVL), 3.92–3.78 (m, 16H), 3.70 (t, 6.4 Hz, 4H), 3.66–3.57 (m, 8H), 3.53–3.41 (m, 8H), 3.32 (t, 7.2 Hz, 4H), 3.09 (brs, 4H), 2.66 (t, 7.9 Hz, 4H), 2.41–2.27 (m, nH, PmVL), 2.23–2.03 (m, 2nH, PmVL), 1.87 (s, 6H), 1.75–1.66 (m, nH, PmVL), 1.57–1.48 (m, nH, PmVL), 1.05–0.93 (m, 3nH, PmVL).

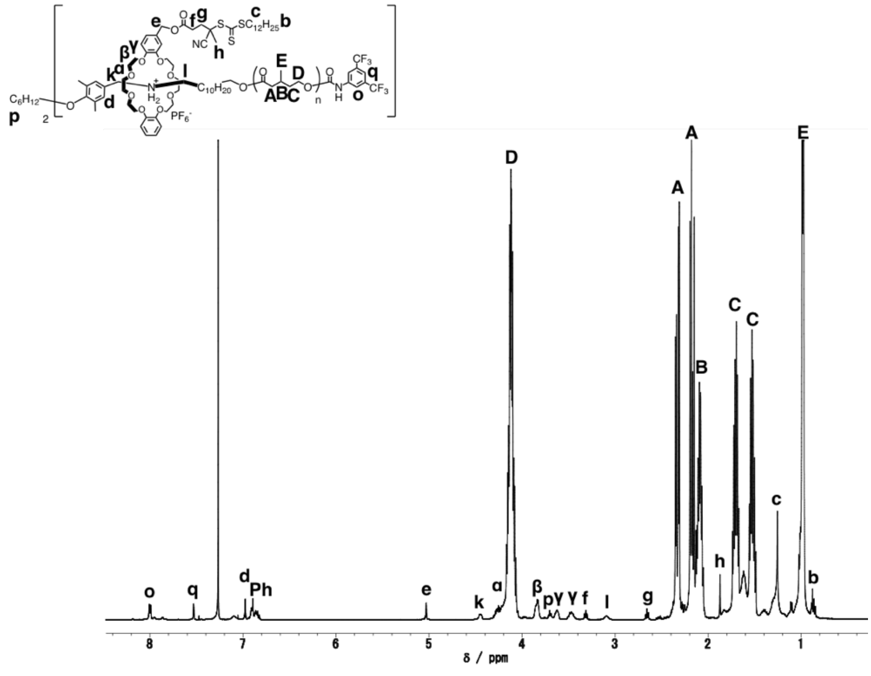

Supplementary Figure 18-1. ^{1}H-NMR spectrum of **Linear polymer** (500 MHz, $CDCl_3$, 298 K).

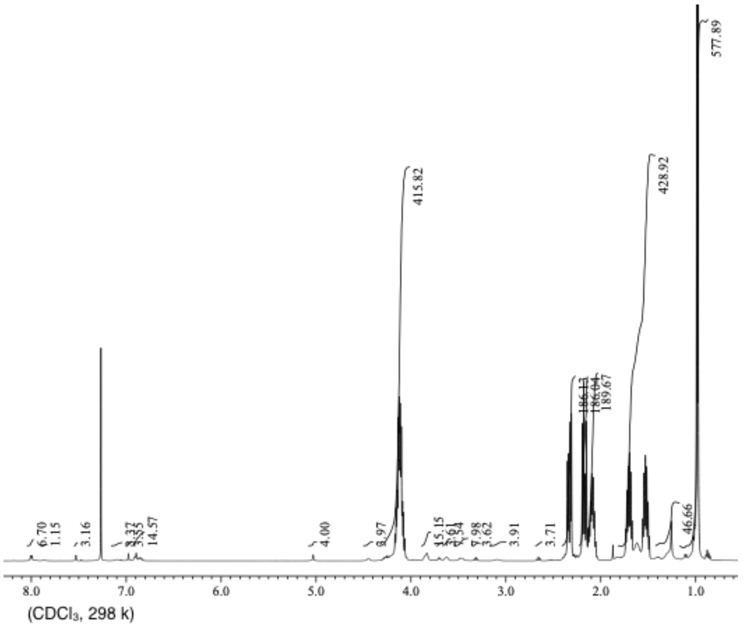

Supplementary Figure 18-2. ^{1}H-NMR spectrum with integration values of **Linear polymer** (500 MHz, $CDCl_3$, 298 K).

Synthesis of A_2B_2

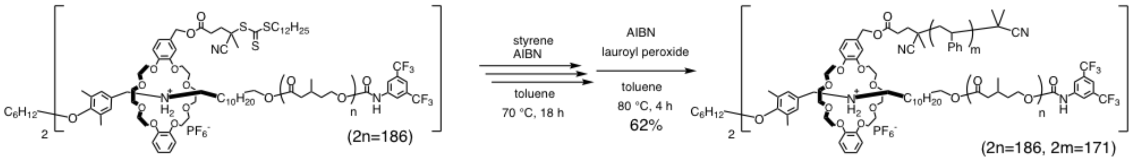

Linear polymer **A_2B_2**

A mixture of **Linear polymer** (261 mg, 0.011 mmol), styrene (0.69 mL, 6 mmol), and AIBN/toluene (4.8 mg/mL, 70 μL, 2 μmol) was degassed three times by freeze-pump-thaw cycles, sealed, and heated at 70 °C. After 18 h, the crude polymer was obtained by pouring into iced hexane (394 mg).

A mixture of the crude polymer (394 mg), lauroyl peroxide (17 mg, 0.042 mmol), AIBN (69 mg, 0.42 mmol), and toluene (4 mL) was degassed three times by freeze-pump-thaw cycles, sealed, and heated at 80 °C. After 4 h, the crude polymer was obtained by pouring into iced hexane (560 mg). Purification by preparative GPC (column: YMC-GPC T-60000) yielded A_2B_2 (281 mg, 62%) as colorless film.

^{1}H-NMR (500 MHz, $CDCl_3$): 7.30–6.20 (PS aromatics), 4.32–4.01 (m, 2nH, PmVL), 2.40–2.30 (m, nH, PmVL), 2.22–2.04 (m, 2nH, PmVL), 2.0–1.2 (PmVL+PS), 1.03–0.96 (m, 3nH, PmVL).

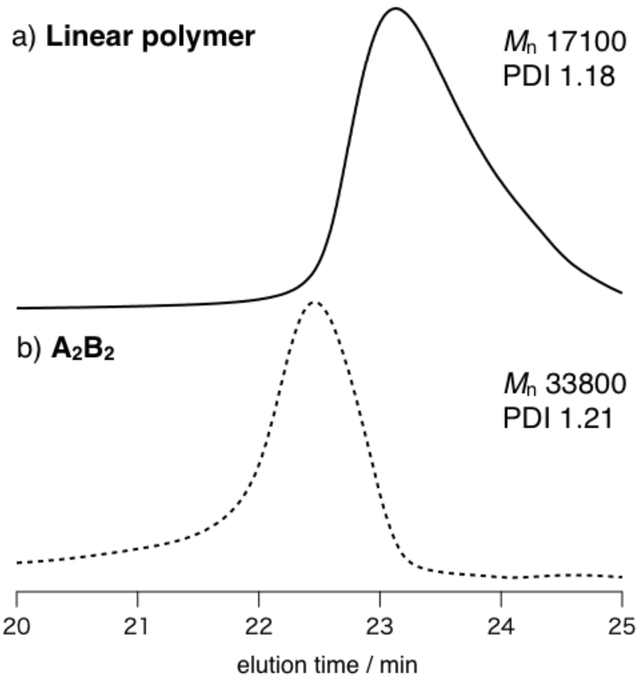

Supplementary Figure 19. GPC traces of (a) **Linear polymer** and (b) **A_2B_2** ($CHCl_3$, RI, PS standards).

2-2) Topology transformation from A_2B_2-star to ABA-linear

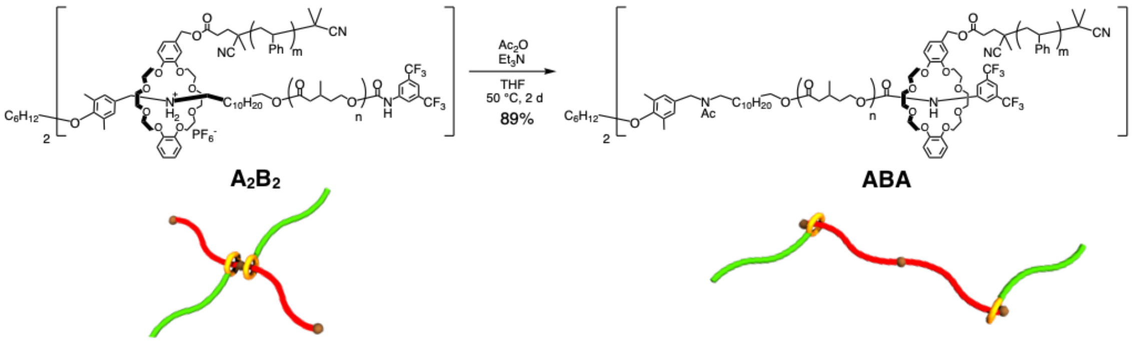

A mixture of **A_2B_2** (140 mg), Ac_2O (16 μL), Et_3N (46 μL), and THF (2 mL) were degassed by sonication under Ar atmosphere and heated at 50 °C. After 2 days, the reaction was quenched by pouring into iced hexane, followed by reprecipitation into iced hexane/EtOH (90/10), to yield **ABA** (125 mg, 89%) as colorless film.

^{1}H-NMR (500 MHz, $CDCl_3$): 8.82 (s, 2H), 7.92 (s, 4H), 7.29 (s, 2H), 7.26–6.29 (PS aromatics), 4.26–4.03 (m, 2nH, PmVL), 2.36–2.30 (m, nH, PmVL), 2.20–2.03 (m, 2nH, PmVL), 2.0–1.2 (PmVL+PS), 1.03–0.93 (m, 3nH, PmVL).

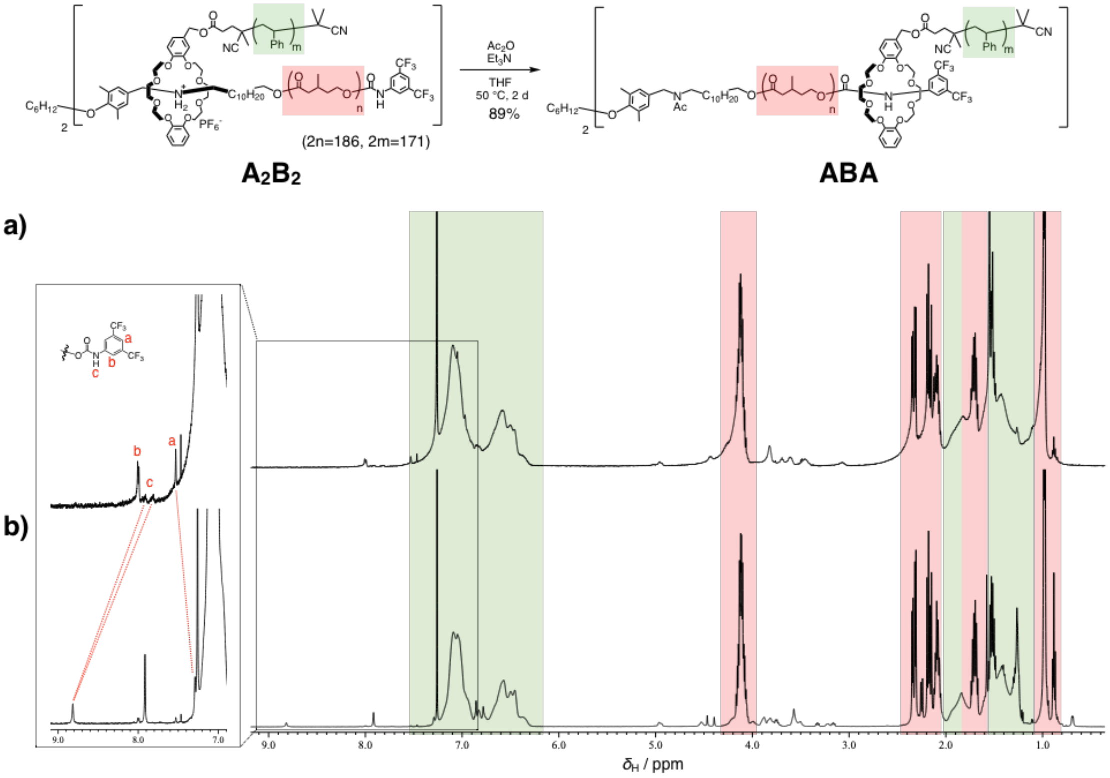

Supplementary Figure 20-1. ^{1}H-NMR spectrum of **A_2B_2** (upper) and **ABA** (lower)

(500 MHz, $CDCl_3$, 298 K).

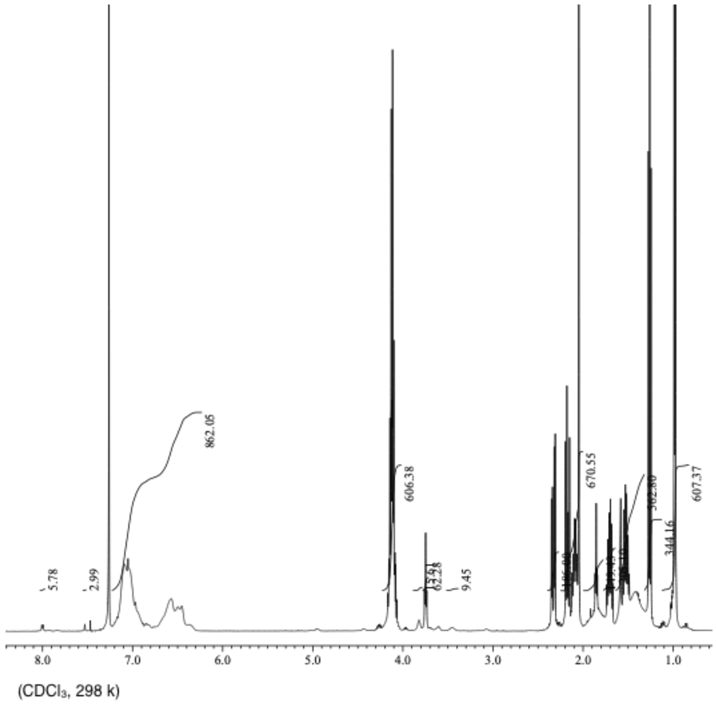

Supplementary Figure 20-2. ¹H-NMR spectrum with integration values of **A$_2$B$_2$** (500 MHz, $CDCl_3$, 298 K).

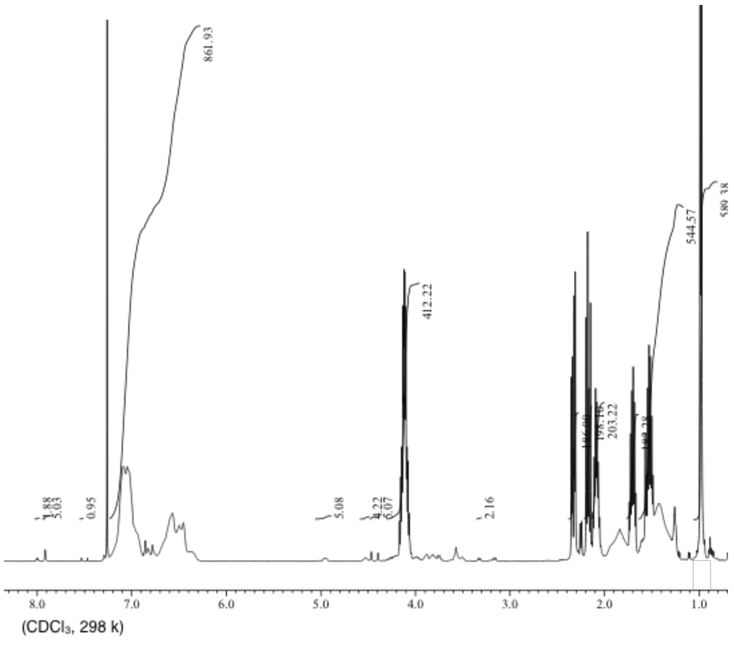

Supplementary Figure 20-3. ¹H-NMR spectrum with integration values of **ABA** (500 MHz, $CDCl_3$, 298 K).

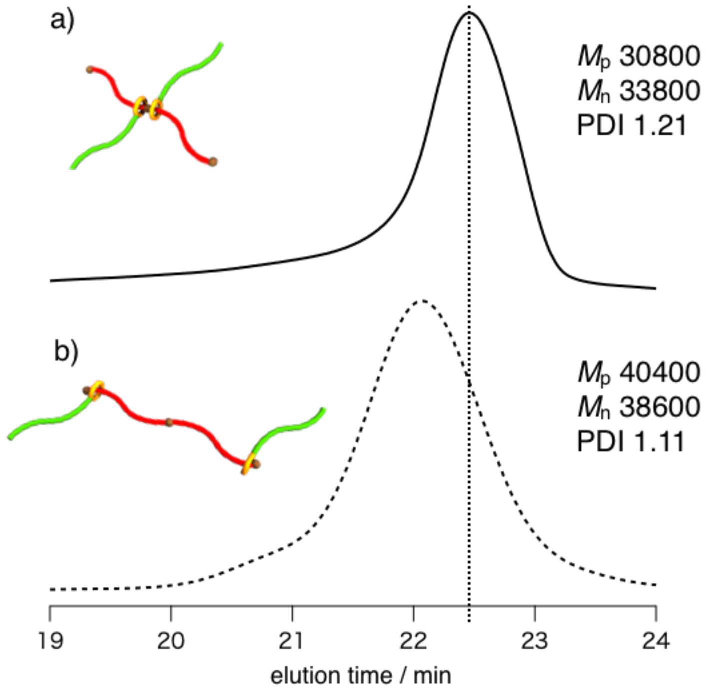

Supplementary Figure 21 GPC traces of a) $\mathbf{A_2B_2}$ and b) **ABA** ($CHCl_3$, RI, PS standards).

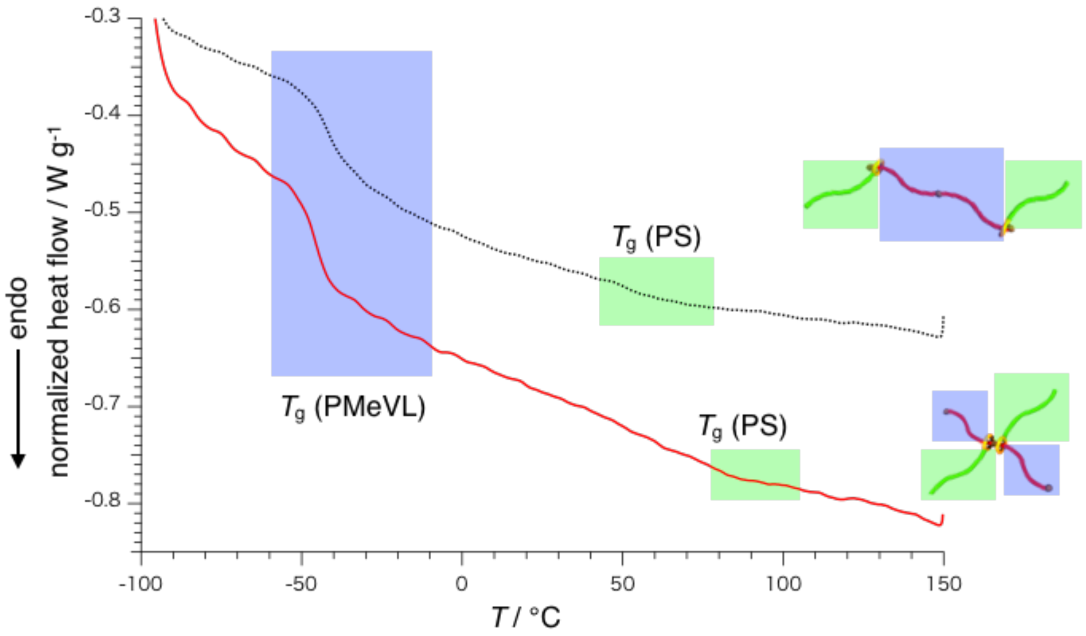

Supplementary Figure 22. DSC traces of $\mathbf{A_2B_2}$ (solid, red) and **ABA** (dot, black) (2nd heating, 20 °C/min).

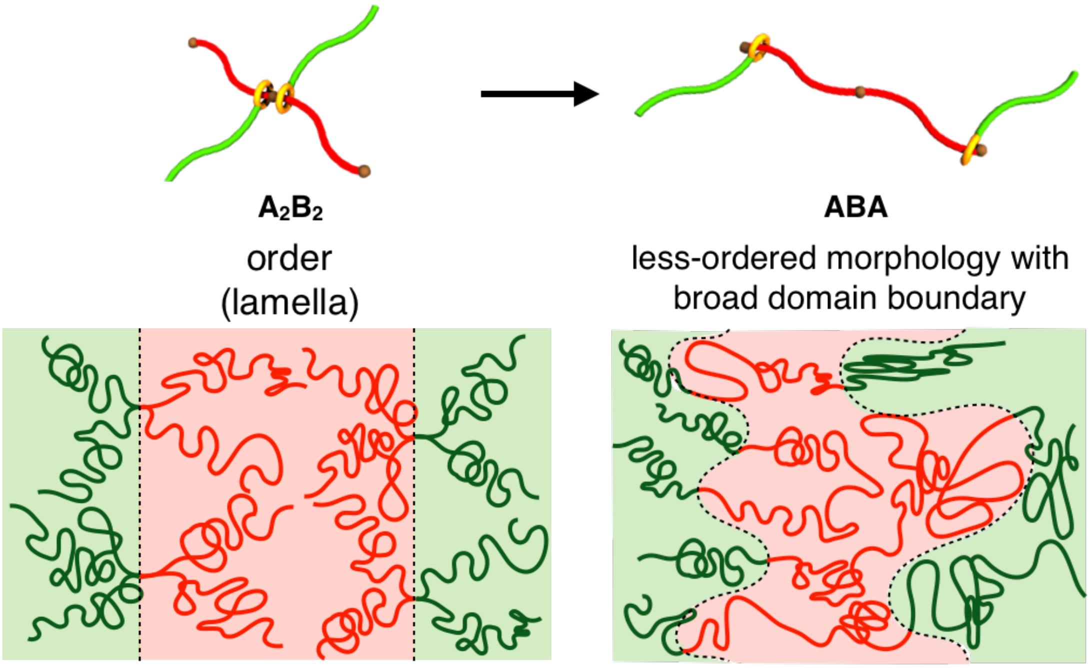

Supplementary Figure 23. Ordered (lamellar) and less-ordered morphology caused by topology transformation

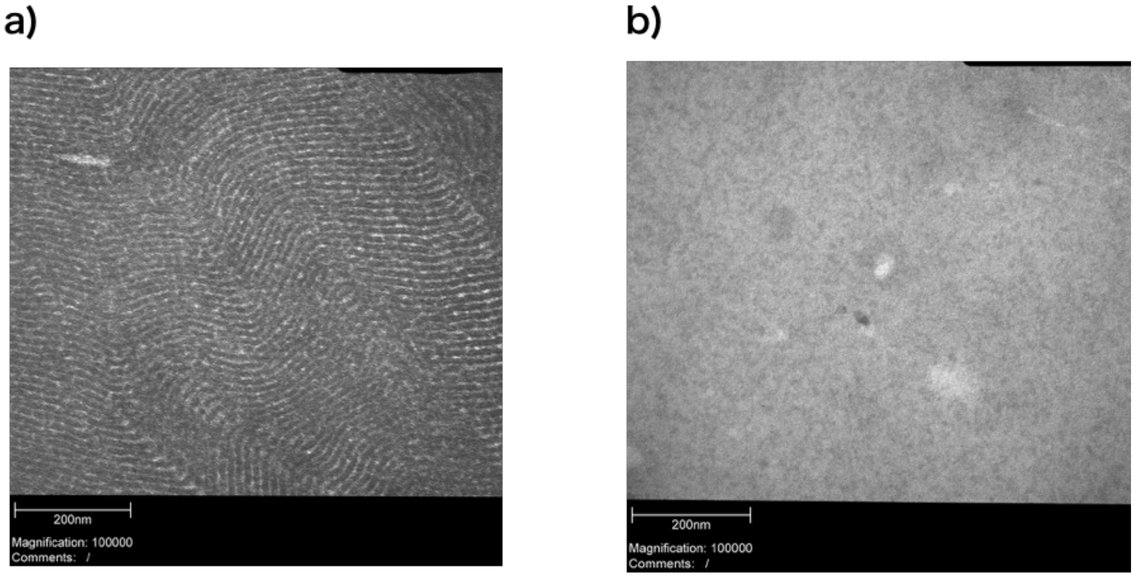

Supplementary Figure 24. TEM images a) before (**A_2B_2**) and b) after (**ABA**) topology transformation. Ordered (lamellar) and less-ordered morphology caused by topology transformation.

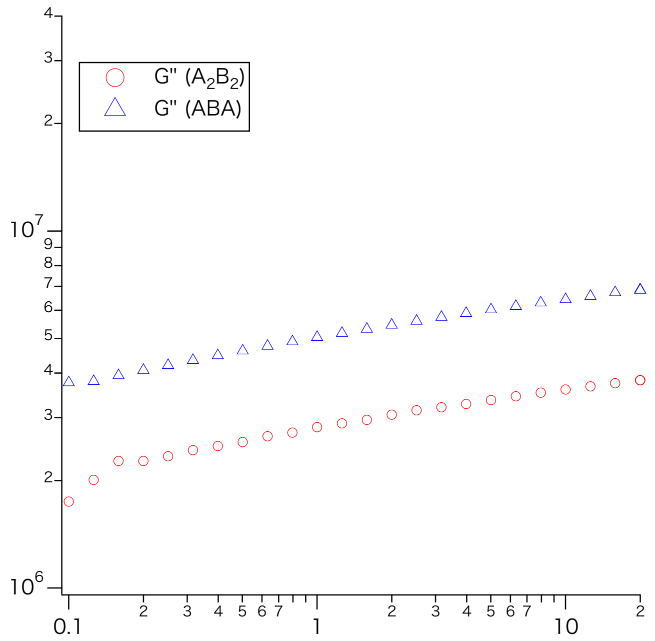

Supplementary Figure 25. Shear rheology profiles (G") before (**A_2B_2**) and after (**ABA**) the topology transformation at 303 K.

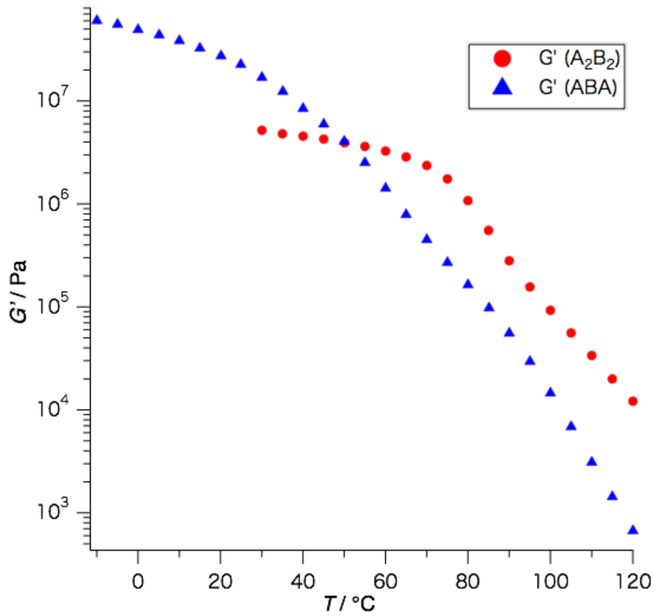

Supplementary Figure 26. Shear rheology profiles (G′) before (● **A_2B_2**) and after (▲ **ABA**) the topology transformation as a function of temperature (1 Hz).

Supplementary Discussion

Model polymer to confirm defiant structure of macromolecular [2]rotaxane consisting of a PmVL-based polymer chain and a wheel.

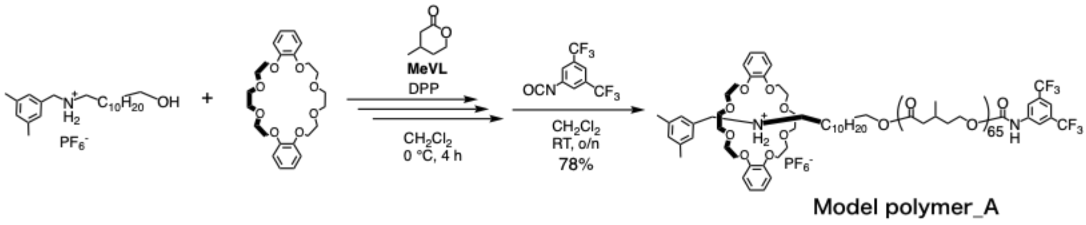

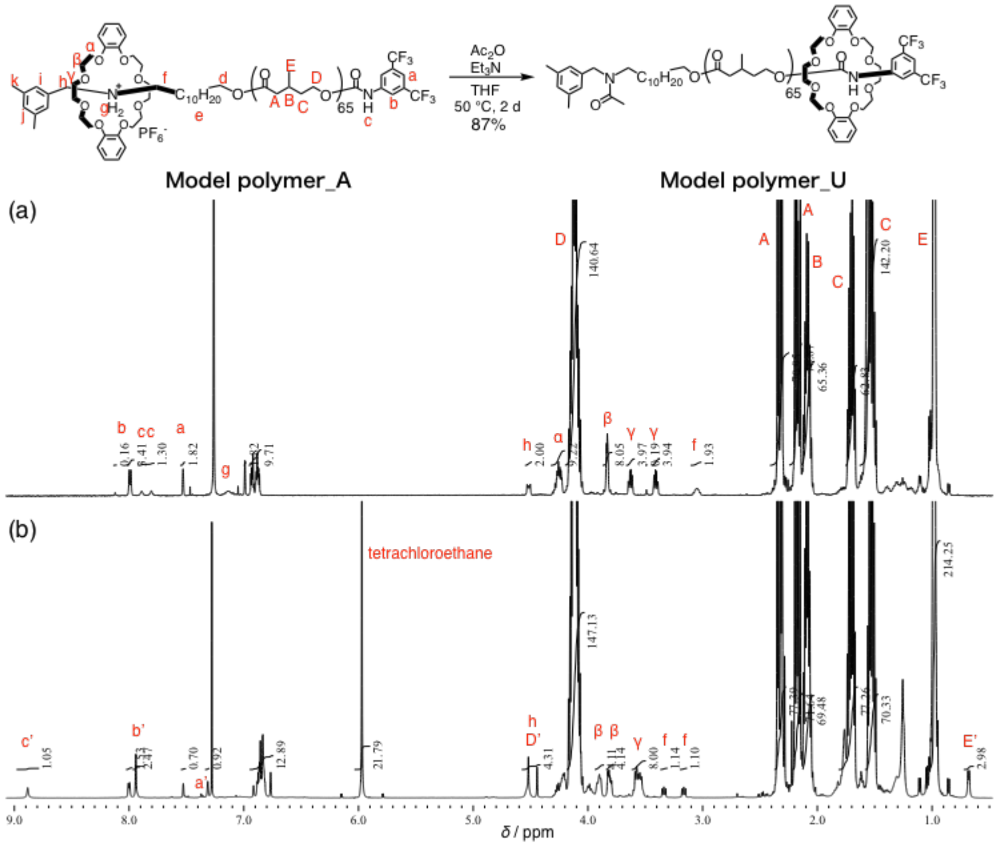

Supplementary Figure 27. ^{1}H-NMR spectra of (a) **Model polymer_A** and (b) **Model polymer_U** (500 MHz, $CDCl_3$, 298 K).

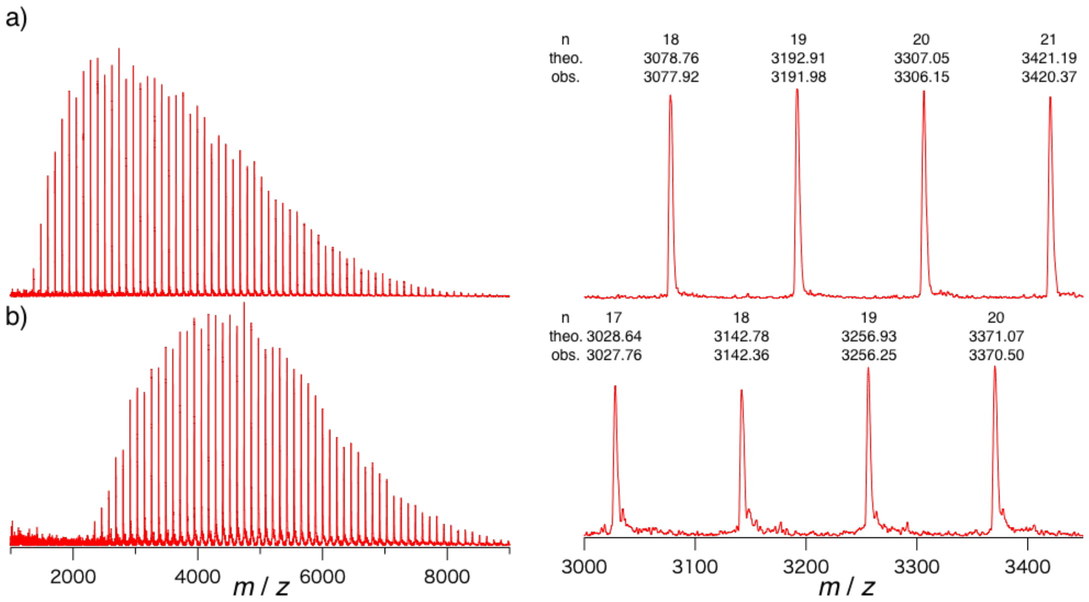

Supplementary Figure 28. MALDI-TOF MS spectra of a) **Model polymer_A** ([M–PF_6]$^+$) and b) **Model polymer_U** ([M+Na]$^+$) (TFANa, dithranol).

Supplementary References

(1) Sato, H., Aoki, D.Takata, T. Synthesis and Star/Linear Topology Transformation of a Mechanically Linked ABC Terpolymer. *ACS Macro Lett.* **5**, 699-703 (2016).

(2) Gurr, P. A., Scofield, J. M. P., Kim, J., Fu, Q., Kentish, S. E.Qiao, G. G. Polyimide Polydimethylsiloxane Triblock Copolymers for Thin Film Composite Gas Separation Membranes. *J. Polym. Sci. Part A: Polym. Chem.* **52**, 3372-3382 (2014).

(3) Marubayashi, H., Asai, S., Sumita, M. Complex Crystal Formation of Poly(L-lactide) with Solvent Molecules. *Macromolecules* **45**, 1384-1397 (2012).

(4) Marubayashi, H., Nojima, S. Crystallization and Solid-State Structure of Poly(L-2-hydroxy-3-methylbutanoic acid). *Macromolecules* **49**, 5538-5547 (2016).

(5) Chen, M., Moad, G.Rizzardo, E. Thiocarbonylthio End Group Removal from RAFT-Synthesized Polymers by a Radical-Induced Process. *J. Polym. Sci. Part A: Polym. Chem.* **47**, 6704-6714 (2009).

(6) Schneiderman, D. K.Hillmyer, M. A. Aliphatic Polyester Block Polymer Design. *Macromolecules* **49**, 2419-2428 (2016).
